# Statin-Related Myotoxicity: A Comprehensive Review of Pharmacokinetic, Pharmacogenomic and Muscle Components

**DOI:** 10.3390/jcm9010022

**Published:** 2019-12-20

**Authors:** Richard Myles Turner, Munir Pirmohamed

**Affiliations:** Department of Molecular and Clinical Pharmacology, Institute of Translational Medicine, University of Liverpool, Liverpool L69 3GL, UK; munirp@liverpool.ac.uk

**Keywords:** statin, pharmacogenomics, muscle toxicity, mitochondria, prenylation, immune system

## Abstract

Statins are a cornerstone in the pharmacological prevention of cardiovascular disease. Although generally well tolerated, a small subset of patients experience statin-related myotoxicity (SRM). SRM is heterogeneous in presentation; phenotypes include the relatively more common myalgias, infrequent myopathies, and rare rhabdomyolysis. Very rarely, statins induce an anti-HMGCR positive immune-mediated necrotizing myopathy. Diagnosing SRM in clinical practice can be challenging, particularly for mild SRM that is frequently due to alternative aetiologies and the nocebo effect. Nevertheless, SRM can directly harm patients and lead to statin discontinuation/non-adherence, which increases the risk of cardiovascular events. Several factors increase systemic statin exposure and predispose to SRM, including advanced age, concomitant medications, and the nonsynonymous variant, rs4149056, in SLCO1B1, which encodes the hepatic sinusoidal transporter, OATP1B1. Increased exposure of skeletal muscle to statins increases the risk of mitochondrial dysfunction, calcium signalling disruption, reduced prenylation, atrogin-1 mediated atrophy and pro-apoptotic signalling. Rare variants in several metabolic myopathy genes including CACNA1S, CPT2, LPIN1, PYGM and RYR1 increase myopathy/rhabdomyolysis risk following statin exposure. The immune system is implicated in both conventional statin intolerance/myotoxicity via LILRB5 rs12975366, and a strong association exists between HLA-DRB1*11:01 and anti-HMGCR positive myopathy. Epigenetic factors (miR-499-5p, miR-145) have also been implicated in statin myotoxicity. SRM remains a challenge to the safe and effective use of statins, although consensus strategies to manage SRM have been proposed. Further research is required, including stringent phenotyping of mild SRM through N-of-1 trials coupled to systems pharmacology omics- approaches to identify novel risk factors and provide mechanistic insight.

## 1. Introduction

Statins are oral hypolipidaemic drugs and amongst the most widely prescribed medications worldwide [1]; in the United Kingdom (UK) alone, ~7 million patients take a statin [2]. The first agent, mevastatin (ML-236B), was identified from *Penicillium citrinum* [3], but was never marketed due to adverse effects. Lovastatin (LVT), isolated from *Aspergillus terreus*, received its marketing authorisation in 1987 and was the first statin approved [4]. LVT also naturally occurs in certain foodstuffs including red yeast rice [5] and oyster mushrooms [6].

Statins are the first line hypolipidaemic drug class for managing cardiovascular (CV) disease (CVD), although ezetimibe, fibrates, bile acid sequestrants, and parenteral proprotein convertase subtilisin/kexin type 9 (PSCK9) inhibitors are also used in specific situations. In the UK, atorvastatin (ATV) 20 mg and 80 mg daily are the current first line guideline-recommended statins for primary and secondary CVD prevention, respectively [7]. However, due to historic prescribing, simvastatin (SVT) remains the most commonly prescribed statin in the UK, followed by ATV [8].

**Figure 1:**
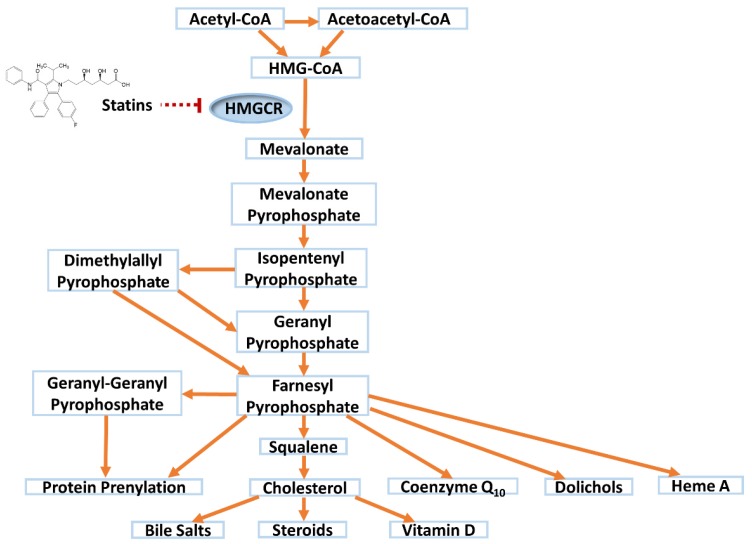
Statin inhibition of the mevalonate pathway.

Statins competitively inhibit 3-hydroxy-3-methylglutaryl-Coenzyme A reductase (HMGCR), the rate limiting enzyme for *de novo* cholesterol synthesis in the mevalonate pathway (Figure 1). In response, a compensatory upregulation in hepatic low-density lipoprotein (LDL) receptor cell surface expression occurs [9], leading to a reduction in circulating LDL cholesterol (LDL-C) by ~30–63%, depending on statin and dose. Statins also reduce triglycerides (~20–40%) and raise high-density lipoprotein-cholesterol (HDL-C) (~5%) to a modest extent [10]. Large meta-analyses of statin randomized controlled trials (RCTs) have concluded that each 1 mmol/L reduction in LDL-C with statin therapy is associated with a 22% reduction in the rate of major CV events (coronary deaths, myocardial infarctions, strokes and coronary revascularisations) [11].

Beyond lowering cholesterol, statins have been associated with a range of beneficial pleiotropic effects including anti-inflammatory, antioxidant and immunomodulatory effects, inhibition of platelet activation, regulation of pyroptosis, and increased plaque stability [12,13,14]. For example, statins mediate a dose-dependent decrease in C-reactive protein [15], may impact renal function [16,17], and attenuate postpartum cardiovascular dysfunction in a rat preeclampsia model [18]. The mechanisms underlying these effects are incompletely understood. However, decreases in other products of the mevalonate pathway following statin-mediated HMGCR inhibition, including isoprenoid intermediates, dolichols, heme A and coenzyme Q_10_ (CoQ_10_,) (Figure 1), are thought to play a role [12].

Seven statins are currently licensed: ATV, fluvastatin (FVT), LVT, pitavastatin (PIT), pravastatin (PVT), rosuvastatin (RVT), and SVT. Statins can be sub-divided into: (i) those administered as the therapeutically inactive lactone (LVT, SVT) versus those administered as active acid statin (ATV, FVT, PVT, PIT, RVT); (ii) those that undergo extensive metabolism by the phase I cytochrome P450 (CYP) system (ATV, FVT, LVT, SVT) versus those excreted predominantly unchanged (PIT, PVT, RVT), and (iii) of the extensively metabolised statins, those primarily biotransformed by CYP3A4/5 (ATV, LVT, SVT) or CYP2C9 (FVT). Table 1 provides an overview of the different statins.

There is notable interindividual variability in response to statin therapy with patients experiencing variable cholesterol lowering efficacy, recurrent CV events [1,15], and a 45-fold variation in statin plasma concentrations [49]. Importantly, a small subset of patients experience statin adverse drug reactions (ADRs), including statin-related myotoxicity (SRM), new-onset diabetes mellitus [50], and elevated liver transaminases [51,52]. Adverse effects on energy levels and exertional fatigue [53] and reduced exercise capacity [54] have been reported, but not confirmed [55]. Similarly, there have been post-marketing case reports of statin-induced memory loss and confusion, although overall, statins are not currently thought to cause cognitive dysfunction [56,57].

It is important to study SRM because, firstly, it can directly harm patients [58,59]. Secondly, despite the unequivocal CVD benefit of statins, statin discontinuation and non-adherence rates are high; ~43% of primary prevention and ~24% of secondary prevention patients become statin non-adherent after a median of ~24 months [60]. Muscle pain increases the likelihood of statin non-adherence and discontinuation [61] which, importantly, increases the risk of major CV events and mortality [62,63].

## 2. SRM Definitions

**Figure 2:**
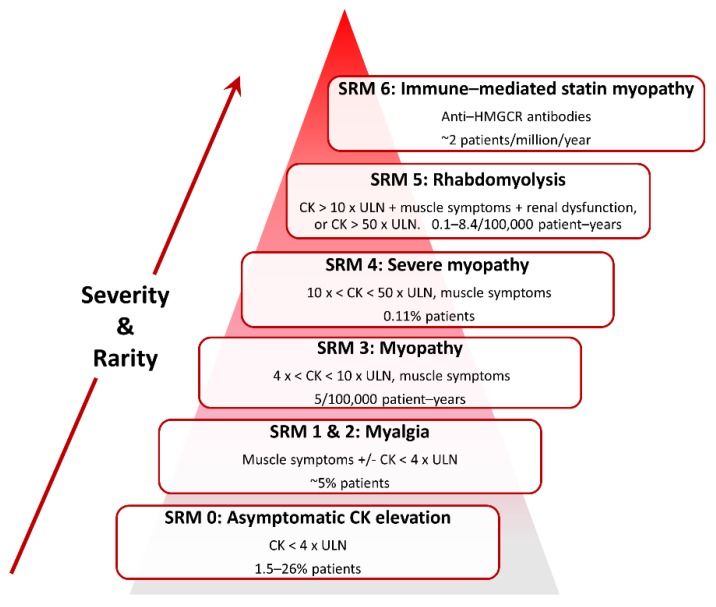
Classification of statin-related myotoxicity phenotypes.

SRM is heterogeneous in presentation (Figure 2) and so case definitions vary between studies. Therefore, a recent effort has standardised nomenclature and classified SRM into seven distinct phenotypic categories [64]:

SRM 0 represents asymptomatic elevations in serum creatine kinase (CK) < 4 × the upper limit of normal (ULN);

SRM 1 and 2 are common myalgias (aches, cramps and/or weakness) with no (SRM 1) or minor CK elevations (< 4 × ULN, SRM 2);

SRM 3 represents increasingly infrequent myopathy with CK > 4 × but < 10 × ULN;

SRM 4 is severe myopathy with CK > 10 × but < 50 × ULN;

SRM 5 constitutes rare but potentially life-threatening rhabdomyolysis with either CK > 10 × ULN, muscle symptoms and renal impairment, or CK > 50 × ULN, and;

SRM 6 consists of very rare anti-HMGCR positive immune-mediated necrotizing myopathy, which persists despite statin cessation [64].

Classification and estimated frequencies are based on Alfirevic et al., 2014 [64], except the myalgia frequency which is from Parker et al., 2013 [65].

Whilst these categories will standardise research, they are perhaps less meaningful as diagnostic criteria in clinical practice. The National Lipid Association (NLA) defines statin intolerance as the “inability to tolerate at least two statins: one statin at the lowest starting daily dose and another statin at any daily dose, due to either objectionable symptoms (real or perceived) or abnormal laboratory determinations, which are temporally related to statin treatment and reversible upon statin discontinuation [66].” The European Atherosclerosis Society (EAS) states “the assessment of statin-associated muscle symptoms includes the nature of muscle symptoms, increased CK levels and their temporal association with initiation of therapy with statin, and statin therapy suspension and re-challenge [67].”

## 3. SRM Clinical Presentation

SRM constitutes the most commonly reported statin adverse event, comprising approximately two-thirds of all adverse events [68]. The most common muscular symptoms are pain, heaviness, stiffness and cramps with or without subjective weakness [58,69]. Symptoms involving leg muscles (thighs, calves) are most frequent, although back, neck, shoulder and generalised muscular symptoms have also been described [58,69]. Tendonitis-associated pain has been reported [58]. Approximately 40% of patients with SRM note a potential trigger; most commonly, unusual physical exertion or a new medication [58]. Muscular pains are intermittent in three quarters of SRM patients, and constant in one quarter [58].

SRM is most common during the first year of treatment [70] with a median time to onset of one month [51]; over 80% of patients report not experiencing similar symptoms before statin treatment [58]. The muscular symptoms in the majority of SRM cases (~70–80%) are sufficiently intense to disrupt everyday activities [58,69]; this includes statin persistence and so, can present as MACE. The rarer severe myopathies and rhabdomyolysis can directly lead to hospitalisation.

## 4. SRM Frequency

Amongst licensed statins, the frequency of SRM appears highest with SVT, followed by ATV, and is lowest with FVT [58]. However, the true incidence of SRM is uncertain, occurring in 1.5–5% of participants in RCTs (relative to placebo groups) [71], compared to ~10–33% in observational studies [61,72]. This variability is potentially attributable to a range of factors, including different myotoxicity definitions and follow up procedures, lead-in periods, inclusion of different patient groups, and treatment blinding [73]. There is consensus that statins increase the risk of severe myopathy and rhabdomyolysis [50]. Of note, cerivastatin (CVT) was voluntarily withdrawn in 2001 because of 52 cases of fatal rhabdomyolysis [74]. However, the variability in reported SRM rates has sparked significant disagreement and controversy over the underlying benefit-risk profile of statins, particularly in patients at the lower end of the CVD risk spectrum [75].

The greater difficulty lies in determining the aetiology of the commoner milder musculoskeletal symptoms, and in particular, whether they are attributable to a statin and/or concurrent condition(s) (e.g., viral illnesses). On the one hand, the frequency of muscle-related adverse events did not differ between patients on ATV 10 mg daily or placebo in the large double-blind ASCOT-LLA RCT (*n* = 10,180), but became significantly more common in patients taking ATV 10 mg daily (1.26% per annum) compared to placebo (1.00% per annum) in the subsequent open label non-blinded extension phase [76]. This observation was attributed to the nocebo effect. On the other hand, a six-month double-blind RCT conducted in 420 healthy volunteers administered ATV 80 mg daily or placebo found increased myalgia amongst the subjects on ATV compared to the placebo group (9.4% vs. 4.6%, respectively, *p* = 0.05) [65]. Moreover, N-of-1 (single-patient) placebo-controlled trials involving patients with a history of SRM have reported that ~30–40% experience subsequent muscle-related events only on statin and not placebo [77,78]. This suggests that the muscle symptoms experienced by a third of symptomatic patients are likely statin-induced, whilst the remainder are probably not. The challenge is how to distinguish patients with true SRM from those with myalgia due to other causes.

## 5. SRM Pathogenesis

**Figure 3:**
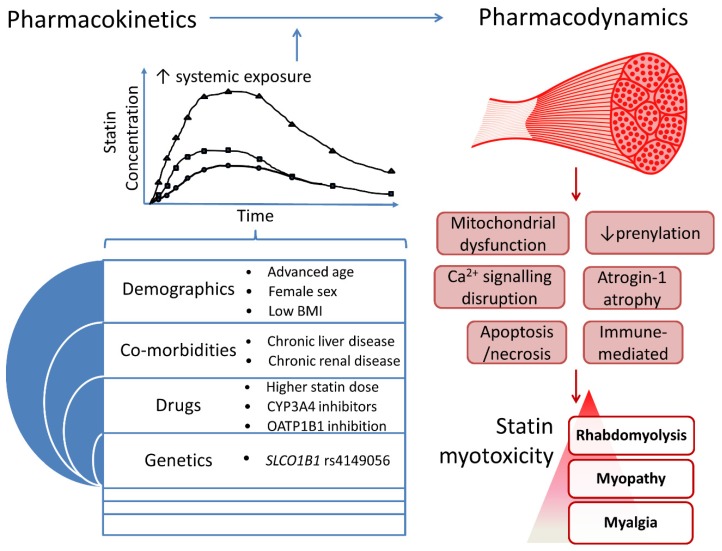
An integrated overview of processes implicated in statin myotoxicity.

Several SRM risk factors have been identified and mechanisms proposed, but there is not yet a unified pathophysiological understanding. Nevertheless, two inter-dependent mechanisms are implicated: 1. increased statin systemic exposure due to clinical and pharmacogenomic factors, which increase skeletal muscle exposure, and 2. intracellular skeletal myocyte entry and disruption of muscle function (Figure 3).

## 6. Factors Associated with Statin Pharmacokinetics and Myotoxicity

The absorption, distribution, metabolism and elimination (ADME) pharmacokinetic (PK) characteristics of the different statins are listed in Table 1. Multiple clinical and pharmacogenomic factors have been associated with statin PK, and a subset also with SRM. These are reviewed below, with particular focus placed on the pharmacogenomic associations.

### 6.1. Clinical Factors

The clinical factors associated with statin PK and SRM are listed in Supporting Information Table S1, and Table 2, respectively. Several, but not all, identified clinical risk factors for SRM are associated with increased statin exposure (Table 2). Increasing dose increases statin exposure. Increasing age correlates with modestly greater statin exposure, except for FVT and RVT [29,43]. Women generally have modestly higher exposure to most statins, except for RVT and ATV. Whilst there is no difference in mean RVT exposure between genders [79], women have modestly lower circulating ATV levels compared to men [21], attributable to higher hydroxylation metabolism. Patients of Asian ancestry have an approximate 1.5–1.9-fold increase in median RVT exposure compared to Caucasian patients [80], and so the US Food & Drug Administration (FDA) recommends that Asian patients start with just 5 mg RVT daily [43]. All statins are predominantly excreted in faeces and so hepatic impairment can result in several fold increased exposure to several statins (e.g., ATV, FVT) [21,29], although the influence on RVT is more modest [81]. The association between alcoholism and SRM [82] may be partially mediated by alcohol-induced hepatic impairment and reduced body mass leading to increased statin exposure, although alcohol itself also causes myopathy [83]. Renal impairment is only associated with increased statin exposure for statins that are at least 10% renally excreted, with little impact on ATV or FVT [21,84]. Thus, the maximum effect of renal impairment is a 3-fold increase in RVT exposure [43]. Importantly, increasing dose, older age, female sex, low body mass index (BMI), liver disease and renal impairment have all been associated with SRM [64].

### 6.2. Pharmacogenomic Factors that Affect Statin Pharmacokinetics

**Figure 4:**
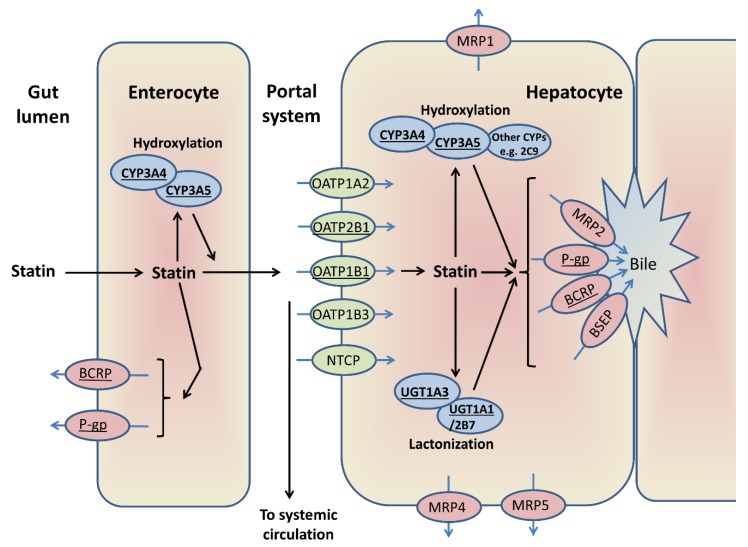
A general schema for statin disposition.

A broad overview of the major enzymes and transporters generally involved in statin disposition is provided in Figure 4. Multiple genes alter statin PK, as summarised in Supporting Information Table S2; key genes are *CYPs*, *UGTs* (uridine 5′-diphospho-glucuronosyltransferases), *SLCO1B1* (solute carrier organic anion transporter family member 1B1) and the efflux transporters *ABCB1* (adenosine triphosphate (ATP)-binding cassette subfamily B member 1) and *ABCG2*, which are reviewed below. Table 3 lists studies that have investigated SRM pharmacogenomics. Overall, of the statin PK genes investigated, only *SLCO1B1* rs4149056 has been consistently associated with SRM.

This figure shows the enzymes and transporters that can be involved in the first pass metabolism of different statins [19,24,38,106,107,108]. ATV, LVT, and SVT are hydroxylated by CYP3A4/5, and FVT by CYP2C9. Statin lactonization is mediated by UDP-glucuronosyltransferases. OATP1B1 is central to the hepatic uptake of statins, although other transporters can be involved. BCRP and/or P-gp are important in the intestinal and biliary efflux of statins, alongside other transporters. The major enzymes/transporters discussed further in this review are underlined.

#### 6.2.1. CYP Phase 1 Hydroxylation

Metabolism is responsible for the clearance of 70% of the top 200 used drugs [142], a subset of 12 of the 57 putatively functional CYPs within the human *CYP* superfamily carry out 75% of drug biotransformations [143], and CYP3A metabolises the largest number of different drugs [142]. ATV, LVT and SVT are themselves extensively metabolised by CYP3A, with greater contributions from CYP3A4 than CYP3A5 [144]. Although no common missense variants are known for *CYP3A4*, the intronic variant, rs35599367 (*22, 522-191C > T), is associated with reduced *CYP3A4* hepatic mRNA and enzymatic activity [145]. CYP3A4*22 increases the formation of non-functional CYP3A4 alternate splice variants with partial intron six retention, specifically in human liver but not small intestine [146]. CYP3A4*22 is present with a minor allele frequency (MAF) of ~5% in Europeans, but is low/rare (~1%) in African and Asian populations [147]. CYP3A4*22 is associated with reduced ATV hydroxylation [148] and ethnicity-restricted increases in SVT/SVT acid concentrations [48]. Although nuclear receptors are highly conserved [149], a single nucleotide polymorphism (SNP) within peroxisome proliferator-activated receptor-α (*PPARA*), rs4253728, has also been associated with reduced human hepatic CYP3A4 protein levels [148] and reduced metabolism of ATV [148] and likely SVT [150].

CYP3A5*3 is a loss of function allele defined by rs776746 (6986G > A), which introduces a cryptic mRNA splice site resulting in a non-functional truncated protein [151], and has MAFs of ~18%, 69% and 94% in African, Asian and European populations, respectively [147], indicating allelic reversal. CYP3A5*3/*3 has been associated with increased SVT and ATV L exposures [152,153].

Increased exposures to LVT [154,155] and SVT [156] have been tentatively reported in association with *CYP2D6* reduction/loss-of-function alleles (e.g., *5, *10, *14). However, in vitro studies have not identified LVT/SVT as CYP2D6 substrates [47,157,158], which puts these *CYP2D6*-LVT/SVT associations into doubt. Carrying CYP2C9*3 has been associated with increased exposures to FVT and PIT, but not RVT or SVT (Table S2). CYP2C9*2 was not associated with FVT exposure [159]. CYP2C9*2 and *3 are both reduction-of-function nonsynonymous variants that reduce xenobiotic metabolism by ~30–40% and ~80–90%, respectively [160]. The MAF of CYP2C9*3 is 7%, 4% and rare in Caucasian, Asian, and African populations, respectively.

Variants in *CYP3A4/5* and *CYP2D6* have been inconsistently associated with SRM or statin tolerability in some candidate gene studies [121,122,123] but not others [85,118,125]. Carrying CYP2C9*2 or *3 may increase the risk of FVT adverse events (mainly myotoxicity), particularly when also receiving a CYP2C9 drug inhibitor [105]. However, all patients in this FVT study were renal transplant recipients [105], and so the generalisability of these findings remains unknown. None of these genes have yet been identified in SRM genome-wide association studies (GWAS) [86,115,141]. Thus, whilst *CYP* genetic variants are linked to altered statin exposure, their relationship with SRM remains uncertain.

#### 6.2.2. UGT1A3 Phase 2 Glucuronidation

The UGT family is involved in phase II drug metabolism and consists of subfamilies UGT1A, UGT2A and UGT2B [161]. UGTs catalyse glucuronidation, typically transforming small lipophilic molecules into more hydrophilic metabolites, which are easier to excrete. Statin lactonization can occur either non-enzymatically at low intestinal pH [162], conceivably via a coenzyme A-dependent process [163], or via an unstable acyl glucuronide intermediate that undergoes spontaneous cyclization to a lactone analyte [164]. Statin lactone species are considered more myotoxic than their acid counterparts [165]. Depending on the statin, UGT1A3, 1A1 and UGT2B7 can be involved in acyl glucuronidation [19]. However, UGT1A3 has been consistently shown to have the highest in vitro statin lactonization rates [19]. UGT1A3*2 is associated with increased UGT1A3 hepatocyte protein expression and *2/*2 volunteers have higher exposures of both ATV lactone and 2-hydroxy ATV lactone [161,166]. The common low expression *UGT1A1* dinucleotide tandem repeat promoter polymorphism, *28, has been associated with both *decreased* area under the ATV lactone concentration-time curve (AUC) [167] and *increased* lactonization [161]; this discrepancy is likely attributable to the extensive linkage disequilibrium within the *UGT1A* locus—for example, between UGT1A1*28 and UGT1A3*2 [161].

*UGT1A1/1A3* variants have been sequenced to investigate CVT myotoxicity, but no association was identified [115]. To date, they have not been included in SRM candidate gene studies, nor identified in SRM GWAS.

#### 6.2.3. SLCO1B1 Influx Transporter

*SLCO1B1*, located on chromosome 12p12.2, encodes organic anion-transporting polypeptide 1B1 (OATP1B1), which is a major hepatocyte-specific sinusoidal influx xenobiotic transporter. The nonsynonymous SNP, rs4149056 (521T > C, p.V174A), in exon five results in decreased intrinsic OATP1B1 transport activity [168]. The rs4149056 MAF is approximately 1%, 8% and 16% in African, Asian and European populations, respectively [147]. Importantly, rs4149056 521CC homozygosity has been associated with increases in statin AUC of 286% (LVT acid) [169], 221% (SVT acid) [170], 208% (PIT) [171], 144% (ATV) [172], 91% PVT [173], and 65% (RVT) [172]. However, rs4149056 has not been associated with FVT [173] or parent LVT [169] and SVT exposures [170].

Importantly, rs4149056 was identified in a seminal GWAS to be strongly associated with myopathy in 85 cases compared to 90 controls, all of whom were on SVT 80mg daily [86]. The odds ratio (OR) for myopathy in 521CC versus 521TT patients was 16.9 (95% confidence interval (CI) 4.7, 61.1), and a gene-dose trend was evident with an OR of 4.5 (95% CI 2.6–7.7) per C allele [86]. In patients on 40 mg SVT daily, the myopathy relative risk remained but was halved to ~2.6 (95% CI 1.3–5.0) per C allele, in keeping with a dose-related ADR [86]. This association between SVT myopathy and rs4149056 has been replicated [89,174] and confirmed in recent large meta-analyses [110,116]. Furthermore, rs4149056 has also been linked to milder adverse outcomes encompassing myalgia, prescription reductions and/or minor biochemical (e.g., CK) elevations indicative of SVT intolerance [69,85,113,175].

In adsition to SVT, historical cases of CVT-related rhabdomyolysis have been associated with rs4149056 [115]. Furthermore, a recent whole-exome sequencing endeavour reported that *SLCO1B1* rs4149056 is associated with statin myopathy (mainly SVT or CVT cases), which reached multiple testing significance when limited to patients not on a fibrate; however, no novel rare coding signals were detected [111]. Intriguingly, *SLCO1B1* rs4149056 has been recently associated with RVT myotoxicity (a composite of myalgias to rhabdomyolysis) in Han Chinese patients [109,176], although it was not previously associated with myalgias in patients of European descent receiving RVT [112]. A recent meta-analysis, largely including these studies, further suggested an association between rs4149056 and RVT myotoxicity [116]. Given the increased RVT exposure reported in Asian compared to Caucasian patients, which is partially but not completed explained by *ABCG2* rs2231142 (see Section 6.2.4) [80], Asian patients are perhaps more sensitive to further *SLCO1B1*-mediated increases in RVT exposure.

Overall, it has been suggested that rs4149056 might be relevant for severe myopathy (e.g., CK > 10 × ULN) due to several statins, with an effect size likely greatest for SVT (or LVT) and lowest for FVT, based upon the degree to which the rs4149056 minor C allele increases exposure to each statin [110]. Nevertheless, rs4149056 has not yet been clearly associated with PVT myotoxicity [85,116], and whilst an association between rs4149056 and ATV myotoxicity has been suggested [85,113] or reported [114], several other studies found no evidence [89,110,116,174,176,177,178]. Reasons for ongoing uncertainty regarding the role of rs4149056 in ATV myotoxicity include fewer ATV cases in studies (especially cases on high dose ATV) [89] and ATV appears less intrinsically myotoxic than SVT [165], as well as the impact of rs4149056 on exposure being smaller for ATV than SVT acid [172]. The latter is plausibly because ATV utilises OATP1B3, 2B1 and 1A2, as well as OATP1B1, for hepatocyte uptake [25].

In summary, the influence of rs4149056 on myotoxicity risk is clear for SVT, but incompletely resolved for the other licensed statins. Importantly, the FDA revised the SVT product label to reduce SVT 80 mg use because of the elevated myotoxicity risk [179]. Furthermore, the Clinical Pharmacogenetics Implementation Consortium (CPIC) guidelines recommend a lower SVT starting dose or an alternative statin, alongside consideration of routine CK surveillance, in patients already known to carry at least one 521C allele [180]. The Dutch Pharmacogenetic Working Group (DPWG) has published guidance for *SLCO1B1* rs4149056 and both SVT and ATV [181]. The DPWG SVT guideline first line recommendation is an alternate statin in 521C carriers, whilst the ATV guidance only recommends an alternate statin in 521C carriers with additional SRM clinical risk factors [181].

#### 6.2.4. ABCB1 and ABCG2 Efflux Transporters

*ABCB1* and *ABCG2* are both members of the superfamily of ATP-binding cassette (ABC) transporters and encode the efflux transporters P-glycoprotein (P-gp) and breast cancer resistance protein (BCRP), respectively. Both P-gp and BCRP are located in the apical (luminal) membrane of enterocytes and the canalicular membrane of hepatocytes, as well as other locations including the blood–brain barrier [182] and placenta [183,184]; they have broad substrate specificity.

*ABCB1* has three common SNPs, rs1128503 (1236T-C, synonymous), rs2032582 (missense, 2677T-G) and rs1045642 (synonymous, 3435T-C); TTT homozygotes have ~55–60% increased exposure to both ATV and SVT acid [185]. The *ABCB1* T alleles have been associated with symptom-independent elevated CK levels [117] and muscle symptoms [118,119] in some candidate gene studies, but not with prescribing changes suggestive of statin intolerance [121], nor in SRM GWAS [110,115].

The nonsynonymous *ABCG2* SNP, rs2231142 (421C > A, p.Q141K), has MAFs of 1%, 10–29% and 9% in African, Asian, and European populations, respectively [147]. The 421AA genotype has been associated with a 2.4-fold increased exposure to RVT, ~2-fold increased exposures to ATV, FVT, and SVT, but no increased exposures to PIT or PVT [108]. Interestingly, carrying rs2231142 421A has been associated with an increased risk of myotoxicity with ATV [120], and in renal transplant recipients receiving FVT [105]. Both of these studies were small case control candidate gene studies and have not been confirmed in GWAS, although SRM GWAS analyses have included relatively few FVT cases to date [86,110,111,115].

### 6.3. Drug–Statin Interactions

Drug–statin interactions are common, can lead to several fold increases in statin exposure, and are established SRM risk factors. Ciclosporin is a potent inhibitor of CYP3A4 [186] and several transporters including OATP1B1, OATP1B3, OATP1B2, ABCG2, and P-gp [99,187], and universally increases systemic exposure of all statins (Table S1). Gemfibrozil and its glucuronide metabolite inhibit CYP2C8 and OATP1B1 and increase statin acid levels (except FVT). Importantly, ciclosporin and gemfibrozil are strongly associated with SRM [99]. CYP3A inhibitors (e.g., amiodarone, itraconazole, clarithromycin) consistently increase the systemic exposure of the CYP3A-metabolised statins (ATV, LVT, SVT) and are significant SRM risk factors [98,99]. Similarly, grapefruit juice, which inhibits CYP3A, has been linked to SVT rhabdomyolysis [96]. The novel cytomegalovirus viral terminase inhibitor, letermovir, increased ATV AUC by over 200%, attributable to inhibition of OATP1B1/3 and CYP3A, and is expected to increase exposure to other statins too [188]. Several antiretroviral drugs increase statin exposure through inhibition of CYP3A and/or OATP1B1, including protease inhibitors (e.g., lopinavir, saquinavir, tipranavir) and pharmacokinetic enhancers (e.g., ritonavir, cobicistat) [189]. As stated above, CYP2C9 inhibitors (e.g., fluconazole) may interact with CYP2C9*2 or *3 carriage to increase FVT myotoxicity [105]. Beyond PK interactions, other drugs themselves linked with myotoxicity, including corticosteroids and colchicine, may also augment the risk of SRM [88,190]. In recognition of the importance of these interactions, specific recommendations for the management of clinically significant statin–drug interactions have been published [190].

## 7. Statin Uptake into Skeletal Muscle

Elevated systemic statin exposure plausibly increases intra-myocyte statin concentrations. Statin myocyte entry is likely facilitated by transporters, with statins being substrates for several sarcolemmal transporters. These include OATP2B1, multidrug resistance-associated protein (MRP) 1, MRP4, MRP5 and MCT4 (monocarboxylate transporter-4) [24,191]. Interestingly, the minor allele of the *SLCO2B1* nonsynonymous variant, rs12422149 (935G > A, p.R312Q), has been associated with increased SVT acid plasma clearance in population PK modelling [150], and with statin (mainly SVT) myalgia in a small candidate gene study (*n* = 19) [69]; both of these findings are potentially consistent with increased statin muscle uptake. It is also noteworthy that lipophilic statins (ATV, SVT) preferentially accumulate in skeletal muscle relative to hydrophilic statins (PVT, RVT) [192], which may help explain the greater myotoxicity of lipophilic statins [165]. The tissue distribution of transporters may also partially account for the lack of statin cardiomyotoxicity [191].

## 8. Statin-Induced Myocyte Dysfunction

Several mechanisms of myotoxicity have been proposed, as outlined below. Studies that investigated the role of muscle-related gene variants in SRM are detailed in Table 3.

### 8.1. Exercise

Physical exercise has been reported to trigger and exacerbate SRM [58]. Following the Boston marathon, runners taking a statin had higher CK rises than runners not on a statin [193]. Interestingly, increasing age was associated with higher CK elevations after the marathon only in those on a statin [193]. In professional athletes with hypercholesterolaemia, only 20% could tolerate a statin long term despite re-challenges with alternate statins and doses [194]. Thus, exercise and statins together can potentiate muscle adverse events [195]. Nevertheless, a systematic review has reported that the literature is inconsistent on whether statins objectively reduce exercise capacity and performance [55]. Interestingly, whilst the circulating levels of three muscle-specific microRNAs (miR-1, miR-133a, miR-206) increased after running a marathon irrespective of statin use, the circulating level of a fourth muscle microRNA, miR-499-5p, only increased 24 h after the marathon in runners taking a statin [196]. Follow-up studies in cultured C2C12 myotubes confirmed that extracellular miR-499-5p increases only when carbachol-induced muscle contraction is combined with statin exposure [196]. These observations suggest a role for epigenetics in statin-potentiated muscle injury, and suggest a biomarker for identifying patients with exercise-exacerbated SRM. Nevertheless, this biomarker requires replication. Lastly, these microRNA observations are from marathon runners and not necessarily applicable to more common, moderate exercise. Intriguingly, it has been suggested by some rodent studies that graduated exercise training can improve muscle tolerance to statin exposure [197,198]. Therefore, the findings that exercise to different degrees may either exacerbate or protect against SRM suggests that further work is required in this area to provide patients with clear advice on what to do in terms of exercise and statin use.

### 8.2. Pre-Existing Neuromuscular Disorders

Statin therapy can adversely interact with underlying neuromuscular disorders to exacerbate symptoms in patients with diagnosed disorders, or unmask previously asymptomatic disorders [199]. Clinical conditions exacerbated or unmasked by statin exposure include myasthenia gravis, dermato/polymyositis, inclusion body myositis, motor neuron disease, and MELAS (mitochondrial encephalopathy, lactic acidosis, and stroke-like episodes) [200,201,202]. MELAS is a rare mitochondrial disease generally associated with mutations in *MT-TL1* (mitochondrially encoded tRNA leucine 1, also known as *TRNL1*) and reported patients adversely affected by statin exposure had the *MT-TL1* A3243G mutation [201,202]. In such cases, symptoms (muscle-related or otherwise) often persist after statin cessation [129,203], which is an indication for further investigations in those patients not already known to have a neuromuscular disorder.

Patients with untreated hypothyroidism, which causes hypercholesterolaemia and hypothyroid myopathy, are at an increased risk of SRM. This SRM can resolve following statin discontinuation, or persist until thyroid hormone replacement [204,205].

Several metabolic myopathies have been associated with SRM, and often, patients were asymptomatic and unaware of the myopathy before starting statin treatment [130]. It is thought that these conditions increase susceptibility to SRM through reducing the ability of skeletal muscle to compensate to statin-induced myotoxic effects. Metabolic myopathies with an identified genetic mutation that has subsequently been found in patients presenting with SRM include: adenosine monophosphate deaminase (*AMPD1*) deficiency (formerly myoadenylate deaminase deficiency) [129], carnitine palmitoyltransferase 2 (*CPT2*) deficiency [129], glycogen storage diseases II (Pompe disease; *GAA* deficiency) [133], V (McArdle disease, *PYGM* deficiency) [129] and IX (muscle phosphorylase b kinase (*PHKA1*) deficiency) [131], malignant hyperthermia (*RYR1*, *CACNA1S*) [135,136], recurrent childhood myoglobinuria (*LPIN1* mutation) [134], and type I (*DMPK*) [130] and II (*CNBP*) [132] myotonic dystrophy. In addition, immune-mediated rippling muscle disease presenting after statin exposure has been reported [206], and mitochondrial myopathies presenting as rhabdomyolsis have been unmasked following statin treatment, although mitochondrial genetic mutations were not identified in these cases [130,207].

By way of example, the carrier frequency for McArdle disease was 12-fold higher in a cohort of patients with lipid lowering (predominantly statin)-induced myopathy, compared to general population controls [129]. One patient that developed muscular complaints *only* after CVT was homozygous for *PYGM* 49XX, a genotype of McArdle disease [129]. McArdle disease is an autosomal recessive disease due to complete deficiency of myophosphorylase (PYGM) activity. Myophosphorylase is a cytoplasmic enzyme involved in glycogenolysis; myophosphorylase deficiency limits muscle oxidative phosphorylation most likely due to impaired substrate delivery to mitochondria [208]. The roles of other select myopathy genes (*CPT2*, *RYR1*, *CACNA1S*) are covered in more detail in the relevant sections below. Overall, a background of carrying variants or incomplete penetrance of metabolic myopathies appears to sensitive individuals to statin myotoxicity.

### 8.3. Mitochondrial Impairment

An important role for mitochondrial impairment in SRM is indicated by the case reports [130,202,207] and series [129] that identified underlying mitochondrial dysfunction in patients with (non-resolving) SRM. For example, CPT2 is located within the mitochondrial inner membrane and undertakes oxidation of long-chain fatty acids in mitochondria alongside CPT1. The carrier frequency of *CPT2* variants associated with CPT2 deficiency was higher in SRM patients compared to controls [129]. CPT2 deficiency is an autosomal recessive disorder and a patient with genetically confirmed CP2 deficiency (113LL) was also identified in this study. This patient did have pre-existing symptoms, exacerbated by CVT [129]. Importantly, in vitro transcriptomic analysis has demonstrated that *CPT2* is amongst the top 1% of genes whose mRNA levels are perturbed by 75 drugs (including statins) that can cause rhabdomyolysis [209].

In vitro studies have demonstrated that the statin lactone species are markedly more myotoxic than statin acids, and SVT lactone and FVT lactone are more myotoxic than ATV lactone and PVT lactone [165]. Following ATV re-challenge, patients with previous SRM had higher systemic exposures to ATV lactone and 4-hydroxy ATV lactone (plus increased 2-hydroxy and 4-hydroxy ATV metabolite levels) compared to healthy controls [210]. Lactones have been shown to strongly inhibit (up to 84%) mitochondrial complex III and reduce respiratory capacity within in vitro myoblasts [192]. Furthermore, Q_0_ of complex III was identified in silico to be an off-target binding site for statin lactones (but not statin acids) [192]. These observations were verified in muscle biopsies from SRM patients, in which complex III enzyme activity was reduced by 18% [192]. Interestingly, CVT lactone showed the greatest degree of complex III inhibition [192], in keeping with its pronounced rhabdomyolysis risk [74]. In contrast, a recent study in healthy male volunteers found no major differences in mitochondrial respiratory capacity after two weeks of daily SVT (80 mg) or PVT (40 mg). However, this study did find a trend for increased sensitivity to the complex I-linked substrate, glutamate, after SVT treatment, which might be an early indicator of adverse effects on skeletal muscle [211]. Moreover in primary human skeletal muscle cells (myotubes), SVT has been shown to impair respiration at mitochondrial complex I, increase mitochondrial oxidative stress through generation of reactive oxygen species (mitochondrial superoxide and hydrogen peroxide), and result in myotube apoptosis [212]. Other studies have reported that statin exposure does not affect the mitochondrial membrane potential [192,213,214], and so statins are unlikely to act as a mitochondrial uncoupler. Lastly, a recent in vitro study has reported that CVT-induced muscle mitochondrial dysfunction is associated with decreased intracellular miR-145 and increased pro-apoptotic gene expression (*APAF1*, *CASP10*); enforced miR-145 expression reduced the apoptotic cell population. However, this study was in a rhabdomyosarcoma cell line and requires replication [215].

Overall, the evidence strongly supports mitochondrial dysfunction in SRM pathogenesis. However, further clarity and unification on the mechanisms are required.

### 8.4. HMGCR Pathway Mediated Effects

Statin inhibition of HMGCR perturbs the mevalonate pathway (Figure 1). Whilst this perturbation has been linked to possible beneficial pleiotropic effects [12], importantly, the decreases in CoQ_10_, protein prenylation, and cholesterol itself have all also been implicated in SRM.

#### 8.4.1. Coenzyme Q_10_ Depletion

CoQ_10_ is an important cofactor in mitochondrial respiration [216]. Primary CoQ_10_ deficiency is a clinically and genetically heterogeneous condition, considered autosomal recessive, and has been associated with isolated myopathy, encephalopathy, nephrotic syndrome, cerebellar ataxia and severe infantile multisystemic disease [217]. In patients on statins, reduced circulating CoQ_10_ is routinely observed [216] and a modest decrease in muscle CoQ_10_ has been suggested in some [218] but not other studies [211,219]. *COQ2* encodes para-hydroxybenzoate-polyprenyl transferase, and defective *COQ2* has been associated with primary CoQ_10_ deficiency, which can improve with early CoQ_10_ supplementation [220]. *COQ2* variants, and in particular rs4693075 (1022C > G), have been investigated; some candidate gene studies [114,127], but not others [89], have reported an association with SRM. Importantly, a recent meta-analysis of RCTs found that CoQ_10_ supplementation likely does not reduce SRM, although larger trials are required to confirm this conclusion [221]. One possible explanation for this null result is that the Q_0_ site of mitochondrial complex III is involved in the transfer of electrons from CoQ_10_ to cytochrome *c*, and Q_0_ is also the off-target binding site for statin lactones (Section 8.3) [192]. Therefore, statins appear to both reduce circulating CoQ_10_ *and* compete for its pharmacodynamic (PD) target; thus CoQ_10_ supplementation alone may insufficiently counteract both statin actions.

#### 8.4.2. Reduced Protein Prenylation

Farnesyl pyrophosphate (FPP) and geranylgeranyl pyrophosphate (GGPP) are both downstream metabolites of mevalonate, and facilitate post-translational prenylation of multiple proteins [222]. GGPP, rather than FPP, is consistently implicated in in vitro statin myotoxicity [213,223,224,225]. Experimental evidence has suggested that the statin-mediated decrease in GGPP reduces myotube ATP levels [213], blocks prenylation of small GTPases including Rab [213,224,226] and RhoA [225], induces atrogin-1 expression [227], and stimulates apoptosis [213,225]. The possible pathways that culminate in apoptosis include RhoA mis-localisation from the cell membrane to the cytoplasm (examined in fibroblasts) [225], inhibition of AKT (protein kinase B) phosphorylation and activation [228] likely via both statin-mediated ATP depletion through mitochondrial dysfunction and loss of Rab1 activity [229], and dose-dependent caspase-3 activation [225].

#### 8.4.3. Cholesterol Depletion

The depletion of cholesterol itself has been posited as an aetiological factor in SRM pathogenesis. Slight skeletal muscle damage has been found by electron microscopy in skeletal muscle biopsies from asymptomatic statin-treated patients, with a characteristic pattern involving T-tubular system breakdown and sub-sarcolemmal rupture [230]; cholesterol extraction could reproduce these findings in vitro in skeletal muscle fibres [230]. Nevertheless, although statins inhibit de novo cholesterol production in C2C12 myotubes, total intracellular cholesterol pools remain unchanged [219]. Furthermore, the PCSK9 inhibitors, alirocumab and evolocumab, even more potently reduce LDL-C than statins, but do not currently appear to increase muscle-related adverse events [231,232]. This suggests that SRM is more statin-specific than cholesterol-specific.

### 8.5. Atrogin-1 Upregulation

The F-box protein, atrogin-1, is a tissue-specific ubiquitin protein E3 ligase that appears central to mediating the proteolysis associated with muscle atrophy observed in multiple diseases, including diabetes and renal failure [233]. Atrogin-1 expression is significantly higher in muscle biopsies from patients with SRM, and atrogin-1 knock down in zebrafish embryos prevented LVT-induced myotoxicity [234]. Moreover, it has been shown that SVT-mediated inhibition of AKT phosphorylation is associated with upregulation of atrogin-1 mRNA [229].

### 8.6. Calcium Signalling Disruption

RYR1 (chromosome 19) and RYR3 (chromosome 15) mediate the release of stored calcium ions from skeletal muscle sarcoplasmic reticulum, and thereby, play a role in triggering muscle contraction [235]. Deleterious *RYR1* variants are associated with anaesthesia-induced malignant hyperthermia, central core disease [236] and multi-minicore disease [237]. *CACNA1S* encodes the alpha-1 subunit of the L-type calcium channel (the dihydropyridine receptor) which associates with RYR1 in skeletal muscle, and *CACNA1S* mutations are associated with malignant hyperthermia and hypokalaemic periodic paralysis. Importantly, disease-causing mutations or variants in *RYR1* and *CACNA1S* have been found to be more frequent in statin myopathy patients than controls [135,136]. Furthermore, muscle biopsies from patients with SRM express significantly higher *RYR3* mRNA and have more severe structural damage, including intracellular T-tubular vacuolisation, than both statin-naïve and statin tolerant controls [238].

A recent study that examined statin-treated human and rat muscle tissue identified that statin treatment causes dissociation of the stabilising protein, FKBP12, from RYR1 in skeletal muscle, and this is associated with increased unwarranted calcium release sparks [197]. In vitro evidence further suggested that uptake of calcium by mitochondria stimulates reactive oxygen/reactive nitrogen species generation that, in turn, act on RYR1 to maintain and/or exacerbate this calcium release from the sarcoplasmic reticulum. Nevertheless, although the calcium sparks were associated with upregulation of pro-apoptotic signalling markers (caspase-3 and the proportion of TUNEL positive nuclei), statin treatment had no impact on muscle force production [197], and so other susceptibility factors are likely required for myotoxicity to manifest. In rats, running wheel exercise normalised FKBP12-RYR1 binding, which suggests a mechanism by which graduated exercise may improve statin tolerance. Statin treatment also had minimal effect on calcium sparks from statin-treated rat cardiac tissue [197].

Lastly, the intronic variant, rs2819742 (1559G > A), in *RYR2* (chromosome one) was suggestively associated with CVT severe myopathy by GWAS [115]. The minor A allele was associated with reduced myopathy risk (OR 0.48, 95% CI 0.36, 0.63, *p* = 1.74 × 10^−7^) [115]. Similarly, a small candidate gene study (*n* = 19) also identified the G allele of *RYR2* rs2819742 to be significantly more common in statin myalgia cases to statin-tolerant controls, in keeping with the GWAS finding [69]. However unlike RYR1/RYR3, RYR2 is expressed mainly in cardiac muscle tissue and deleterious *RYR2* mutations are associated with ventricular arrhythmias [239]. Therefore, the relevance of *RYR2* rs2819742 to SRM remains unclear.

### 8.7. Glycine Amidinotransferase (GATM)

A genome-wide expression quantitative expression loci (eQTL) analysis in lymphoblastoid cell lines derived from 480 clinical trial subjects identified rs9806699 as a cis-eQTL for *GATM*, which interacted with in vitro SVT exposure such that it was a significantly stronger eQTL under SVT-exposed versus control conditions [126]. GATM is involved in creatine synthesis, and phosphorylation of creatine by CK is a major mechanism for muscle energy storage. The *GATM* locus was associated with a reduced incidence of statin myopathy in two separate populations (combined SVT, ATV, PVT) with a meta-analysis OR for rs1719247 of 0.60 (95% CI 0.45–0.81, *p* = 6.0 × 10^−4^) [126]. Several subsequent SRM studies of SRM have not replicated this finding [110,111,240,241,242], although a recent candidate gene study of RVT myotoxicity in Han Chinese patients found a similar marginal protective effect of the *GATM* rs9806699 minor allele (*p* = 0.024) [109]. The lack of replication raises questions about the role of *GATM* in SRM; functional studies of *GATM* in human primary muscle cells may help resolve the discordant results.

### 8.8. Immunologically-Mediated Statin Myopathy

#### 8.8.1. LILRB5

A GWAS of serum CK levels found strong signals with the muscle CK (*CKM*) gene and a missense variant, rs12975366 (D247G), within leukocyte immunoglobulin-like receptor subfamily B member 5 (*LILRB5*) [243]; these results were replicated in statin users and non-users [243]. Subsequently, D247 homozygosity has been associated with an increased risk of statin intolerance (a definition not reliant on CK), and replicated in two of three separate cohorts of patients with either myalgia on RVT, or statin myopathy (meta-analysis OR 1.34, 95% CI 1.16-1.54, *p* = 7 × 10^−5^) [139]. CK levels were included as a covariate, where appropriate. Subgroup analysis in the included RCT interestingly showed that, whilst D247 homozygosity was associated with myalgia with both placebo and RVT, those carrying 247G only had an increased myalgia risk if on RVT. Thus, whilst D247 homozygosity might confer an overall greater risk of myalgia, statin-induced myalgia appears associated with 247G. A randomized cross-over experimental medicine study to further investigate this drug-gene interaction is being undertaken [244]. Although the exact aetiology is unknown, the immune system is involved in the repair of skeletal muscles and the influx of Foxp3 + T regulatory cells are crucial to muscle regeneration [245]; interestingly, *LILRB5* D247 may associate with *FOXP3* expression [139].

#### 8.8.2. HLA-DRB1*11:01

Interestingly, several research groups previously noted that symptoms and CK elevation in a few patients with SRM persist and/or progress after statin discontinuation, and furthermore, these patients benefit from immunosuppressive therapy [246,247,248]. These features are consistent with an autoimmune phenomenon. In 2011, it was reported that these patients, as well as a minority without prior statin exposure (less than 10% in myopathy patients ≥ 50 years old), are positive for anti-HMGCR autoantibodies [249]. Muscle biopsies often show necrotizing myopathy with minimal lymphocytic infiltration [137,250], and so anti-HMGCR positive myopathy is recognised as a distinct subtype of immune-mediated necrotizing myopathy [251]. Pharmacogenomic studies have provided further evidence of an autoimmune aetiology. Importantly, HLA-DRB1*11:01 has been significantly associated with anti-HMGCR positive myopathy [137,138], and the ORs for the presence of HLA-DRB1*11:01 in anti-HMGCR myopathy white or black patients, compared to controls, have been estimated to be ~25 and ~57, respectively [138]. HLA-DRB1*11:01 has also been associated with the development of anti-Ro antibodies in neonatal lupus. Although the underlying aetiology of immune-mediated necrotizing myopathy remains incompletely resolved, a potential role for anti-HMGCR in its pathogenesis is suggested: muscle HMGCR expression is upregulated in anti-HMGCR positive myopathy patients [249], circulating anti-HMGCR levels correlate with CK concentration and disease activity [252], and anti-HMGCR can impair muscle regeneration and induce muscle atrophy [253].

### 8.9. Pain Perception

A family history of muscular symptoms with or without statin exposure increases the risk of SRM [58,69]. A candidate gene study in 195 statin-treated patients, of whom 51 experienced at least probable myalgia, found that rs2276307 and rs1935349 in the 5-hydroxytryptamine (5-HT, serotonin) receptor genes (*HTR*), *HTR3B* and *HTR7*, respectively, were significantly associated with myalgia score [140]. This suggests that variants that may produce individual differences in pain perception might play a role in statin-taking patients’ reports of muscle pain [140]. No 5-HT-related candidate SNP was associated with serum CK level [140], suggesting that the associations are with pain perception rather than the extent of muscle breakdown. Nevertheless, these associations have not been replicated in SRM GWAS analyses [86,110,115], although these GWAS analyses used CK elevation (muscle breakdown) within their case definition [86,110,115]. Moreover, these associations were not identified in a small (*n* = 19) candidate gene study of statin myalgia [69]. Overall, an assessment in a larger cohort with statin myalgia cases will help finalise the relevance of these findings.

### 8.10. Muscle Transcriptomics

The multifaceted and complex pathogenesis of SRM has been underlined by a recent study that compared muscle transcriptomic profiles in 26 cases of strictly phenotyped statin myalgia undergoing statin re-challenge (75% re-developed muscle symptoms) to 10 statin-tolerant controls, with most taking SVT [69]. A robust separation in skeletal muscle differentially expressed genes was found that highlighted the roles of mitochondrial stress, cell senescence and apoptosis, localised activation of a pro-inflammatory immune response, and altered cell and calcium signalling mediated by protein prenylation and Ras-GTPase activation, in statin myalgia [69]. For example, the insulin/IGF/PI3K/AKT signalling network was the top perturbed canonical pathway. Within this network, calmodulin (*CALM*) was upregulated [69]. CALM is a calcium sensing protein that interacts with RYR1, and the calcium-calmodulin complex inhibits RYR1 [254]. Alternatively, inositol 1, 4, 5-triphosphate receptor 2 (*ITPR2*) can medicate calcium release from the sarcoplasmic reticulum [255], and was downregulated within this network [69]. These differential patterns of regulation likely influence calcium signalling and are conceivably an adaptive response to the increased RYR1-mediated calcium release sparks identified following statin-dependent FKBP12 dissociation from RYR1 (described in Section 8.6) [197]. The two most strongly upregulated genes were antisense RNA to the HECT domain E3 ubiquitin protein ligase 2 (*HECTD2-AS1*) and uncoupling protein 3 (*UCP3*). HECTD2 is pro-inflammatory, whilst UCP3 is a mitochondrial anion carrier protein posited to protect against oxidative stress [69]. Although atrogin-1 ubiquitin E3 ligase was not differentially expressed in this study, several genes of the ubiquitin ligase pathway (including HECTD2) did feature prominently in this study [69].

### 8.11. Vitamin D

The vitamin D family are a group of fat-soluble secosteroids that are instrumental in the regulation of calcium and phosphate levels, and bone mineralisation; the most important forms in humans are cholecalciferol (vitamin D_3_) and ergocalciferol (vitamin D_2_). The major natural source of vitamin D is via the conversion of 7-dehydrocholesterol (endogenously synthesised from cholesterol) to cholecalciferol by UV-B light, although ergocalciferol and cholecalciferol can also be obtained from plant and animal-derived dietary sources, respectively [256]. Vitamin D is inactive and so undergoes sequential hydroxylation, first to 25-hydroxycholecalciferol/25-hydroxyergocalciferol, which are the major circulating forms but also inactive, and then to 1, 25-dihydroxycholecalciferol (calcitriol)/1, 25-dihydroxyergocalciferol (collectively 1, 25(OH)2D) that constitute the biologically active vitamin D species [256]. 1,25(OH)2D acts through the vitamin D receptor, which is located in multiple tissues including bone, kidney, intestine, parathyroid glands and skeletal muscle, to mediate genomic and faster non-genomic actions [256,257].

There is controversy regarding the impact of statins on vitamin D level [258]. Nevertheless, 1, 25(OH)2D induces CYP3A4 [259,260] and consistent with this finding, the oral availability and systemic exposure of the CYP3A4 substrate, midazolam, trends higher in winter than summer [261]. Similarly, vitamin D supplementation reduces ATV exposure [262]. However, paradoxically, low vitamin D levels may blunt lipid-lowering response to ATV, perhaps because vitamin D derivatives can also inhibit HMGCR [256]. Vitamin D deficiency causes osteomalacia/rickets, as well as muscle weakness and myopathy. Importantly, a meta-analysis has confirmed that plasma vitamin D levels are significantly lower in statin-treated patients with myalgia, compared to those without [93]. Furthermore, several (non-randomized) clinical studies have reported that vitamin D supplementation effectively reduces incident SRM in patients previously statin intolerant undergoing re-challenge, particularly when previously low vitamin D levels are documented to become normalised [91,263,264,265]. Based on these findings, a double-blind adequately powered RCT is now required.

## 9. Management of SRM

As statins are widely prescribed, mild SRM is commonly encountered in clinical practice, although statin rhabdomyolysis remains rare. For a patient presenting with SRM, an initial CK level should be taken. During the consultation, the EAS recommend an evaluation of clinical risk factors for SRM (Table 2), other causes of muscular complaints (e.g., polymyalgia rheumatica), and to review the indication for statin therapy, particularly in those at low CVD risk [67]. The benefits and risks of continuing, temporarily suspending, and discontinuing statin treatment need to be weighed up. Additional patient counselling involves discussion about the nocebo effect and complimentary therapeutic lifestyle changes (e.g., smoking cessation, blood pressure control, adopting the Mediterranean diet) [67,266]. There is no gold-standard diagnostic method nor a validated questionnaire for SRM, although a myalgia clinical index score has been proposed by the NLA [267]. Nevertheless, the majority of patients that discontinue statin treatment after a statin-related event can subsequently tolerate some form of statin therapy if re-challenged [268]. In patients with SRM and an ongoing statin indication, temporary statin withdrawal is often appropriate, followed by one or more statin re-challenges (post washout), which can aid causality assessment. Re-challenges can use the same statin (at same dose), an alternate statin at usual dose, lower doses (with potential up-titration), and/or intermittent (non-daily) dosing using a high intensity statin with a long half-life (e.g., ATV, RVT) [67]. The aim should be to treat with the maximum tolerated dose required for the indication [7]. Patients should also be informed that any statin at any dose lowers CVD risk [7]. Nevertheless, whilst less intense approaches such as intermittent dosing are tolerated in at least 70% of patients, they lead to a variable and likely lower proportion of patients reaching LDL-C goals [269], which should also be discussed. In those that do not reach LDL-C goals, non-statin lipid lowering therapy can be considered in combination with the maximally tolerated statin dose or as monotherapy; available options include ezetimibe, a fibrate, or PCSK9 inhibitor. If considering fibrate therapy, fenofibrate is preferred, and gemfibrozil should be avoided because of its interaction with statins to increase rhabdomyolysis risk [67]. Alirocumab and evolocumab have demonstrated cardiovascular benefit in clinical outcomes trials [231,270]. Moreover, in statin-intolerant patients, these PCSK9 inhibitors are tolerated by > 80%, reduce LDL-C by 45–56%, and have fewer muscular adverse events than ATV re-challenge [232,271]. Nevertheless, the costs of PCSK9 inhibitors remain high. As a consequence, this often limits their use to select patients with severe dyslipidaemia [272], and is prohibitive for broader adoption in CVD prevention [273].

Recently, bempedoic acid has shown promise in patients unable to tolerate more than low-dose statin therapy. Bempedoic acid is a novel oral agent under development that inhibits ATP citrate lyase, and a phase 3 RCT showed it reduced LDL-C by 28.5% more than placebo, without a greater rate of muscle-related events. Of note, ATP citrate lyase is upstream of HMGCR, but bempedoic acid is a prodrug that requires very-long chain acyl-CoA synthetase-1 (ASCV1L) for bioactivation. ASCV1L is expressed predominantly in the liver and so it is plausible that the limited active bempedoic acid in muscle will reduce any potential for myotoxicity. It is also noteworthy that a large multicentre implementation initiative is pre-emptively genotyping patients starting one of 39 drugs for over 45 pharmacogenomic variants, and prospectively determining the incidence of ADRs compared to standard care [274]. For patients starting SVT (or ATV) with at least one *SLCO1B1* rs4149056 minor allele, the DPWG recommendations are provided to them and their healthcare team [274].

## 10. Conclusions

Despite the development of PCSK9 inhibitors, and ongoing development of novel promising therapeutics including bempedoic acid [275] and inclisiran [276], the undoubted efficacy, affordability, availability, and widespread experience with statins ensure they will likely remain the cornerstone of lipid lowering therapy for the foreseeable future. Thus, understanding and mitigating SRM remains clinically relevant. The majority of SRM is mild and ceases quickly after statin cessation. In patients in whom symptoms persist, a non-statin related diagnosis is most likely, although an unmasked metabolic myopathy, or immune-mediated anti-HMGCR positive myopathy, should also be considered. SRM can cause direct patient harm, and the links between muscular symptoms, suboptimal statin utilisation, and increased MACE are clear [61,62,63]. Several factors that increase systemic statin exposure are associated with SRM, including higher statin dose, advanced age, drug-drug interactions and, for SVT, *SLCO1B1* rs4149056. Increased systemic statin (lactone) exposure, in turn, predisposes to downstream deleterious effects on skeletal muscle. The most important appear to be mitochondrial dysfunction, calcium signalling disruption and reduced prenylation, whose sequelae include atrogin-1 mediated atrophy, apoptosis, and likely reduced immune-mediated muscle regeneration.

At present, our potential to predict SRM is limited. The parsimonious ‘QStatin’ model for statin moderate-severe myopathy [70] has been developed, which includes new statin use, ethnicity, co-morbidities (liver disease, hypothyroidism, diabetes mellitus) corticosteroids, age and BMI, although its area under the receiver operator curve of ~0.7 is modest [88]. The implementation of *SLCO1B1* rs4149056 testing [274] may help improve predictive power. Whilst the association between HLA-DRB1*11:01 and anti-HMGCR positive myopathy is notably strong, HLA-DRB1*11:01 will likely be insufficient to predict this condition alone given its rarity, but HLA-DRB1*11:01 may have utility in excluding the diagnosis.

Overall, further research is critically needed to identify, validate and integrate novel risk factors for the different SRM phenotypes to improve predictive capability and harmonise understanding of SRM pathogenesis. We propose that the integration of strict clinical phenotyping to identify statin-induced myalgia through the N-of-1 trial paradigm [277], with systems pharmacology omics-based approaches, should be beneficial. Replication of the miR-499-5p and miR-145 signals is needed. The interactions between exercise and vitamin D status with statin use warrant further study. Increased research is also needed into the gut microbiome, as it has recently been shown to be significantly perturbed by statins [278,279] and might module statin response [280]. Much has been done; much work remains.

## Figures and Tables

**Table 1:** Pharmacokinetic properties of the different statins.

Drug Property	Atorvastatin	Cerivastatin	Fluvastatin	Lovastatin	Pitavastatin	Pravastatin	Rosuvastatin	Simvastatin
Year approved	1996	1997 to 2001	1993	1987	2009	1991	2003	1991
Generic available	Yes	No	Yes	Yes	No	Yes	No	Yes
Daily dose (mg)	10–80	0.2–0.3	20–80	10–80	1–4	10–80	5–40	10–40
Equipotent dose (mg)	20	-	>80	80	4	80	5	40
Marketed drug form	Acid	Acid	Acid	Lactone	Acid	Acid	Acid	Lactone
log P (N-octanol/H_2_O partition coefficient)	1.11 (lipophilic)	1.70 (lipophilic)	1.27 (lipophilic)	1.70 (lipophilic)	1.49 (lipophilic)	−0.84 (hydrophilic)	−0.33 (hydrophilic)	1.60 (lipophilic)
Oral absorption (%)	30	>98	98	31	80	37	50	65–85
Bioavailability (%)	14	60	29	<5	51	17	20	5
Effect of food on bioavailability	Decrease	No effect	Decrease	Increase	No effect	Decrease	No effect	No effect
Time to C_max_ (hours)	1–2	2–3	2.5–3	2	1	1–1.5	3–5	1–4
Protein binding (%)	≥98	>99	98	>95	>99	~50	88	95
Volume of distribution	381 L	0.3 L/Kg	25	-	148 L	0.5 L/Kg	134 L	233 L
Extent of metabolism	High	High	High	High	Low	Low	Low	High
CYPs that metabolise statin acid form	CYP3A CYP2C8 ^†^	CYP2C8 CYP3A	CYP2C9 CYP2C8 ^†^ CYP3A ^†^	CYP3A	CYP2C9 CYP2C8 ^†^	CYP2C9 CYP3A ^†^	CYP2C9 CYP2C19 ^†^ CYP3A ^†^	CYP3A CYP2C8 ^†^
CYPs that metabolise statin lactone form	CYP3A	CYP3A	CYP3A	CYP3A	CYP3A CYP2D6 ^†^	Not known	CYP3A CYP2C9 ^†^ CYP2D6 ^†^	CYP3A
UGTs involved in lactonization of statin acid form	UGT1A1 UGT1A3 UGT2B7	UGT1A3	Not known	UGT1A1 UGT1A3	UGT1A3 UGT2B7	None identified	UGT1A1 UGT1A3	None identified
Transporters for parent statin	OATP1B1, BCRP, MRP1, 2, 4, NTCP, P-gp, OATP1A2, 1B3, 2B1	OATP1B1, BCRP	OATP1B1, 1B3, 2B1, BCRP	OATP1B1, P-gp	OATP1B1, 1B3, BCRP, MRP2, NTCP, P-gp	OATP1B1, 1B3, 2B1, BSEP, BCRP, MRP2, P-gp; OAT3 in renal elimination	OATP1B1, BCRP, BSEP, MRP1, 2, 4, 5, P-gp, OATP1A2, 1B3, 2B1, NTCP; OAT3 in renal elimination	BCRP, P-gp (SVT acid: OATP1B1)
Metabolites formed	2-OH ATV, 4-OH ATV, ATV L, 2-OH ATV L, 4-OH ATV L	M-1 acid, M-23 acid, CVT L, M-1 L, M-23 L	5-OH FVT, 6-OH FVT, N-deisopropyl FVT, FVT L	LVT acid, 6-OH LVT acid	PIT L	6-epi PVT, 3α-OH PVT, PVT L, 3α-OH PVT L	N-desmethyl RVT, RVT L	SVT acid, 3′,5′-dihydrodiol, 6′-exomethylene & 3-OH acid metabolites
Elimination t_1/2_ (h)	14	2–3	3	2–5	12	1–3	19	2–3
Faecal excretion (%)	98	70	90	83	79	70	90	60
Renal excretion (%)	<2	30	5	10	15	20	10–28	13
References	[19,20,21,22,23,24,25]	[19,25,26,27,28]	[22,25,29]	[19,22,25,30,31]	[19,22,25,32,33]	[22,25,34,35,36,37,38]	[19,22,24,25,39,40,41,42,43,44]	[19,22,25,45,46,47,48]

ATV = atorvastatin; BCRP = breast cancer resistance protein; BSEP = bile salt export pump; CVT = cerivastatin; CYP = cytochrome P450; FVT = fluvastatin; L = lactone; LVT = lovastatin; M-1 = demethylation cerivastatin metabolite; M-23 = hydroxylation cerivastatin metabolite; MRP = multidrug resistance-associated protein; NTCP = sodium-taurocholate co-transporting polypeptide; OATP = organic anion-transporting polypeptide; -OH = hydroxy; P-gp = P-glycoprotein; PIT = pitavastatin; PVT = pravastatin’ RVT = rosuvastatin’ SVT = simvastatin; UGT = uridine 5′-diphospho-glucuronosyltransferase. ^†^ = denotes enzymes with a minor contribution to the known statin metabolism. Drug-metabolising adult CYP3A consists of CYP3A4 and variable CYP3A5 expression, dependent on *CYP3A5* genotype. The underlined transporters are considered particularly important to the disposition of the statin.

**Table 2:** Clinical risk factors of statin-related myotoxicity.

Category	Risk Factor	Reference
**Demographics**		
	Advanced age (>80 years old)	[51,82]
	Female gender	[51,85]
	Low body mass index	[73,82]
**Ethnicity**		
	Black African	[70]
	Caribbean	
**Co-morbidities**		
	Alcohol abuse	[82]
	Chronic kidney disease	[51,82,86,87]
	Chronic liver disease	[70,88]
	Diabetes mellitus	[88,89]
	Hypertension	[90]
	Hypothyroidism	[88]
	Vitamin D deficiency	[91,92,93]
**Personal/family factors**		
	Physical exercise	[58,94,95]
	Personal or family history of muscle pain	[58]
**Diet**		
	Grapefruit juice (CYP3A inhibition)	[96]
**Drugs**		
	Higher statin dose	[70,73,97]
	Corticosteroids	[88]
	CYP3A inhibitors (particularly for ATV, LVT, SVT)—e.g., amiodarone, ciclosporin, clarithromycin, erythromycin, protease inhibitors (e.g., indinavir, ritonavir)	[98,99,100,101,102,103,104]
	CYP2C9 inhibitors ^†^ (for FVT)—e.g., fluconazole	[105]
	OATP1B1 inhibition—e.g., gemfibrozil, ciclosporin	[99]

Adapted from Alfirevic et al., 2014 [64]. ^†^ = in renal transplant patients and limited to the subgroup carrying CYP2C9* 2 or * 3.

**Table 3:** Pharmacogenomic investigations of statin-related myotoxicity.

Study	Design	Genes	Variants	Statin	*N*	Endpoint	Main Results
**Statin Pharmacokinetics**							
Bai 2019 [109]	Co, CG	*SLCO1B1*	rs4149056 (521T > C, p.V174A)	RVT	758	Muscle symptoms +/− ↑CK	-OR 1.74 (95% CI 1.18–2.57), *p* = 0.0052 -No associations for *ABCB1*, *ABCG2*, *CYP2C9*, *SLCO1B3* -*GATM*—see ‘Muscle-related’ section below
Carr 2019 [110]	GWAS, then MA			SVT, CVT, ATV (+ others)	7764	CK > 10 × ULN or rhabdomyolysis	-all statins: OR 2.99 (95% CI 2.34–3.82), *p* = 2.63 × 10^−18^ -SVT: OR 5.91 (95% CI 4.10–8.51), *p* = 1.46 × 10^−21^ -ATV: no clear associations
Carr 2013 [89]	CC, CG			SVT, ATV (+ others)	448	Stop statin & CK > 4 × ULN	-all statins: OR 2.08 (95% CI 1.35–3.23), *p* = 0.005 -SVT: OR 2.13 (95% CI 1.29–3.54), *p* = 0.014 -*COQ2*—see ‘Muscle-related’ section below
Floyd 2019 [111]	MA, WES			SVT, CVT, ATV (+ others)	2552	Muscle symptoms & CK > 4 × ULN	-No genome-wide significant associations -rs4149056 in non-fibrate users secondary analysis: 4.01-fold ↑ risk (95% CI 2.61–6.17), *p* = 5.46 × 10^−11^
Danik 2013 [112]	RCT, CG			RVT	4404	Myalgia	No association detected
de Keyser 2014 [113]	Co, CG			SVT, ATV	1939	Statin dose decrease or switch	-SVT: HR 1.74 (95% CI 1.05–2.88), *p* = 0.033 -ATV > 20 mg: HR 3.26 (95% CI 1.47–7.35), *p* = 0.004 -No associations for SVT or ATV in replication set
Link 2008 [86]	CC, GWAS			SVT	175	CK > 3 × ULN & 5 x baseline, plus ↑ ALT	-SVT 80 mg: OR 4.5 (95% CI 2.6–7.7) -STV 40mg: OR 2.6 (95% CI 1.3–5.0), *p* = 0.004
Puccetti 2010 [114]	CC, CG			ATV, RVT	76	Muscular intolerance (muscle symptoms or ↑ CK or ↑LFTs)	-ATV: OR 2.7 (95% CI 1.3–4.9), *p* < 0.001 -RVT: no association -*COQ2*—see ‘Muscle-related’ section below
Marciante 2011 [115]	CC, CGs & GWAS			CVT	917	Muscle symptoms & CK > 10 × ULN	-OR 1.89 (95% CI 1.40–2.56), *p* = 3.62 × 10^−5^ -No associations for *CYP2C8*, *UGT1A1/1A3* -*RYR2*—see ‘Muscle-related’ section below
Voora 2009 [85]	RCT, CG			ATV, SVT, PVT	452	Stop statin, myalgia, or CK > 3 × ULN	-OR 1.7 (95% CI 1.04–2.8), *p* = 0.03. -Risk highest in patients on SVT. -No apparent association for PVT -No associations for *CYP2C8*, *2C9*, *2D6*, *3A4*
Xiang 2018 [116]	MA, CG			SVT, CVT, RVT, ATV, PVT	11,008	Multiple- myalgia to rhabdomyolysis	-SVT: OR 2.35 (95% CI 1.08–5.12), *p* = 0.032 -CVT: OR 1.95 (95% 1.47–2.57), *p* < 0.001 -RVT: OR 1.69 (95% CI 1.07–2.67), *p* = 0.024 -ATV or PVT: no associations
Elam 2017 [69]	CC, CG	*SLCO1B1*	rs4149056	SVT, ATV, RVT	19	Statin myalgia confirmed by re-challenge	-↑ myalgia with rs4149056 variant allele (*p* = 0.039) -↑ myalgia with rs12422149 variant allele (*p* = 0.001) -*RYR2*—see ‘Muscle-related’ section below
		*SLCO2B1*	rs12422149				
Ferrari 2014 [117]	CC, CG	*SLCO1B1*	rs4149056, rs2306283	ATV, RVT, SVT	66	CK > 3 × ULN, irrespective of symptoms	*-* rs4149056: OR 8.5 (95% CI 1.7–42.3), *p* = 0.001 - rs2306283: OR 0.3 (95%CI 0.06–0.91), *p* = 0.022 -*ABCB1*: OR 4.5 (95% CI 1.4–14.7), *p* = 0.001 -No association for *ABCG2*
		*ABCB1*	1236C > T, 3435C > T				
Fiegenbaum 2005 [118]	Co, CG	*ABCB1*	1236C > T, 2677G > A/T, 3435C > T	SVT	116	Myalgia	-↑ endpoint risk with *ABCB1* variants (*p* < 0.05) -No associations with *CYP3A4, 3A5*
Hoenig 2011 [119]	Co, CG	*ABCB1*	3435C > T	ATV	117	Myalgia	↑ risk carrying T compared to C allele (*p* = 0.043)
Mirosevic Skvrce 2015 [120]	CC, CG	*SLCO1B1*	rs4149056	ATV	130	Adverse reactions (61.7% myotoxicity); myalgia to rhabdomyolysis	-rs4149056: OR 2.3 (95% CI 1.03–4.98), *p* = 0.043 -rs2231142: OR 2.75 (95% CI 1.10–6.87), *p* = 0.03 -No association for CYP3A4*22
		*ABCG2*	rs2231142				
Mirosevic Skvrce 2013 [105]	CC, CG	*ABCG2*	rs2231142	FVT	104	Adverse reactions in renal transplant patients (90.4% myotoxicity)	-rs2231142: OR 4.89 (95% CI 1.42–16.89) -*2 or *3 carriers: OR 2.44 (95% CI 1.05–5.71), *p* = 0.037 -↑ risk of endpoint in CYP2C9*2 or *3 carriers on a CYP2C9 drug inhibitor: OR 6.59, *p* = 0.027
		*CYP2C9*	*2, *3				
Becker 2010 [121]	Co, CG	*CYP3A4*	*1B	SVT, ATV	1239	Statin dose decrease or switch	-SVT/ATV: HR 0.46 (95% CI 0.24–0.90), *p* = 0.023 -SVT only: HR 0.47 (95% CI 0.23–0.96), *p* = 0.039 -No association for *ABCB1*
Frudakis 2007 [122]	CC, CG	*CYP2D6*	*4	ATV, SVT	263	Stop statin due to muscle events	-ATV: OR 2.5 (95% CI 1.5–4.4), *p* = 0.001 -SVT: OR 1.7 (95% CI 0.9–3.2), *p* = 0.067
Mulder 2001 [123]	Co, CG	*CYP2D6*	*3, *4, *5, *2xN	SVT	88	Stop statin	↑ risk with *CYP2D6* variants (RR = 4.7); a gene-dose trend
Wilke 2005 [124]	CC, CG	*CYP3A4/5*	3A4*1B, 3A5*3	ATV	137	Myalgia	No main associations detected
Zuccaro 2007 [125]	CC, CG	*CYPs*	Several	ATV, SVT, PVT (+ others)	100	Muscle symptoms +/− ↑CK	No associations for *CYP2C9*, *2D6*, *3A5*
**Muscle-related**							
Bai 2019 [109]	Co, CG	*GATM*	rs9806699	RVT	758	Muscle symptoms +/− ↑CK	OR 0.62 (95% CI 0.41–0.94), *p* = 0.024
Mangravite 2013 [126]	deQTL CG CCs	*GATM*	rs9806699 rs1719247	SVT	4413	Muscle symptoms & CK > 3 × ULN	-rs1719247 in LD with top deQTL, rs9806699: r^2^ = 0.76 -MA: OR 0.60 (95% CI 0.45–0.81), *p* = 6.0 × 10^-4^
Carr 2013 [89]	CC, CG	*COQ2*	rs4693075	SVT, ATV (+ others)	448	Stop statin & CK > 4 × ULN	*COQ2* rs4693075: no associations
Oh 2007 [127]	CC, CG	*COQ2*	rs6535454 rs4693075	ATV, RVT (+ others)	291	Muscle symptoms + stop statin or CK > 3 × ULN	-rs6535454: OR 2.42 (95% CI 0.99–5.89), *p* = 0.047 -rs4693075: OR 2.33 (95% CI 1.13–4.81), *p* = 0.019
Puccetti 2010 [114]	CC, CG	*COQ2*	rs4693075	ATV, RVT	76	Muscular intolerance (muscle symptoms or ↑ CK or ↑LFTs)	-RVT: OR 2.6 (95% CI 1.7–4.4), *p* < 0.001 - ↑ risk of muscular symptoms and ↑ CK with ATV: OR 3.1 (95% CI 1.9–6.4), *p* < 0.001
Ruano 2011 [128]	CC, CG	*COQ2*	rs4693570	ATV, SVT, RVT (+ others)	793	Myalgia	-*COQ2* rs4693570 (*p* = 0.000041) or *ATP2B1* rs17381194 (*p* = 0.00079) associated with ↓ risk. -*DMPK* rs672348 (*p* = 0.0016) associated with ↑ risk.
		*ATP2B1*	rs17381194				
		*DMPK*	rs672348				
Vladutiu 2006 [129]	CC, CG	*CPT2*	Several	ATV, CVT, LVT, SVT	358	Muscle symptoms; CK ↑ reported	Overall, a fourfold ↑ in the number of mutant alleles (*AMPD1* > *CPT2*/*PYGM*) in cases vs. statin-tolerant controls.
		*PYGM*	R49X, G204S				
		*AMPD1*	Q12X, P48L, K287I				
Tsivgoulis 2006 [130]	CRs, CG	*DMPK*	CTG repeats	PVT, ATV, SVT	4	Muscle symptoms or fatigue & CK ↑	-1 case of each of type 1 myotonic dystrophy (*DMPK*), glycogen storage disease V (muscle histochemical diagnosis), mitochondrial myopathy (muscle biopsy & biochemical diagnosis), and Kennedy disease (*NR3C4*) diagnosed after starting statin and becoming symptomatic
		*NR3C4*	CAG repeats				
Echaniz-Laguna 2010 [131]	Co, CG	*NR3C4*	CAG repeats	SVT, PVT, ATV (+ others)	52	Abnormal EMG & pathological analysis, if muscle features last > 3 months after statin ceased	-5 patients diagnosed with paraneoplastic polymyositis, Kennedy disease (*NR3C4*), glycogen storage disease IX (*PHKA1*), motor neuron disease, and necrotic myopathy of uncertain aetiology
		*PHKA1*	Not specified				
Knoblauch 2010 [132]	CS, CG	*CNBP*	CCTG repeat	ATV, SVT (+ others)	3	Muscle symptoms that last after statin ceased +/− ↑CK	-All 3 cases diagnosed with type II myotonic dystrophy after becoming symptomatic after starting statin treatment
Voermans 2005 [133]	CR, CG	*GAA*	IVS1-13T > G 525del T	SVT	1	Muscle symptoms & CK ↑	-1 case of a compound heterozygote for glycogen storage disease II diagnosed after becoming symptomatic on SVT
Zeharia 2008 [134]	CC, CG	*LPIN1*	sequenced	Unknown	20	Myopathy with ↑CK	In 2 of 6 cases, exonic nucleotide substitutions thought harmful were found, vs. 0 in 14 statin-tolerant controls.
Vladutiu 2011 [135]	CC, CG	*RYR1*	34 mutations	Not specified	493	Muscle symptoms-often last post statin, +/− ↑CK	*RYR1* mutations in 3 of 197 severe & 1 of 163 mild statin myopathies, vs. 0 of 133 statin-tolerant controls
Isackson 2018 [136]	WES	*RYR1*	Pathogenic variants	ATV, RVT, SVT (+ others)	126	Muscle symptoms & CK > 5 × ULN	12 of 76 (16%) of SRM patients had probably pathogenic variants in *RYR1* or *CACNA1S*, which was 4-fold higher than in statin-tolerant controls.
		*CACNA1S*					
Elam 2017 [69]	CC, CG	*RYR2*	rs2819742	SVT, ATV, RVT	19	Statin myalgia confirmed by re-challenge	-↑ myalgia with rs2819742 variant allele (*p* = 0.016) -No associations with *GATM*, *COQ2*, *HTR3B*, *HTR7*
Marciante 2011 [115]	CC, CGs & GWAS	*RYR2*	rs2819742	CVT	917	Muscle symptoms & CK > 10 × ULN	OR 0.48 (95% CI 0.36-0.63), *p* = 1.74 × 10^−7^
**Immune-system related**							
Limaye 2015 [137]	Co, CG	HLA-DRB1*11	Typing to ‘two-digit’ resolution	Not specified	207	Anti-HMGCR antibodies in patients with idiopathic inflammatory myositis or immune-mediated necrotizing myopathy	-Anti-HMGCR antibodies in 19 of 207 myopathy cases -HLA-DRB1*11 more frequent in myopathy patients positive vs. negative for anti-HMGCR antibodies: OR 56.1 (95% CI 5.0–7739), *p* = 0.001 -3 anti-HMGCR positive myopathy patients had high resolution typing and all carried HLA-DRB1*11:01
Mammen 2012 [138]	CC, HLA typing	HLA-DRB1*11	Typing resolution: -Intermediate; -High in *DR11*	Not specified	733	Anti-HMGCR antibodies in patients with myositis/myopathy	-OR for HLA-DRB1*11:01 in anti-HMGCR myopathy patients vs. controls: ~24.5 (*p* = 3.2 × 10^−10^) and ~56.5 (*p* = 3.1 × 10^−6^) in white & black ethnicities, respectively -*HLA-DQA1* and *DQB6* less frequent in white anti-HMGCR positive patients than controls (*p* = 5.5 × 10^−4^, *p* = 2.1 × 10^−5^, respectively)
		*DQA*	Intermediate resolution				
		*DQB*					
Siddiqui 2017 [139]	Co, CG	*LILRB5*	rs12975366	SVT, RVT (+ others)	1034	-1. Non-adherence & ↑CK -2. Statin intolerant & switched ≥ 2 other statins	-1: OR 1.81 (95% CI 1.34–2.45) -2: OR 1.36 (95% CI 1.07–1.73)
**Pain perception**							
Ruano 2007 [140]	CC, CG	*HTR3B*	rs2276307	ATV, SVT, PVT	195	Myalgia	-↑risk for rs2276307 (*p* = 0.007) & rs1935349 (*p* = 0.026) -No associations for *HTR1D*, *2A*, *2C, 3A*, *5A*, *6, SLC6A4*
		*HTR7*	rs1935349				
**Other**							
Isackson 2011 [141]	GWAS	*EYS*	rs1337512, rs9342288, rs3857532	ATV (+ others)	399	Muscle symptoms-often last post therapy, +/− ↑CK	*EYS* SNPs conferred ↑ risk (*p* = 0.0003–0.0008), but did not survive multiple testing correction for GWAS.

CC = case-control study; CG = candidate gene; CI = confidence interval; CK = creatine kinase; Co = cohort study; CR = case report; CS = case series; deQTL = differential expression quantitative trait loci; GWAS = genome-wide association study; HR = hazard ratio; MA = meta-analysis; OR = odds ratio; RCT = randomized controlled trial; WES = whole-exome sequencing. Studies are ordered to preferentially group those that investigated the same gene(s) together.

## Supplementary Materials

The following are available online at https://www.mdpi.com/2077-0383/9/1/22/s1, Table S1: Clinical factors associated with statin exposure, Table S2: Genetic variants associated with statin exposure.
