# Supplementary material for: Statin-Related Myotoxicity: A Comprehensive Review of Pharmacokinetic, Pharmacogenomic and Muscle Components
Source: J Clin Med. 2019 Dec 20;9(1):22. doi: 10.3390/jcm9010022 (PMC7019839; doi:10.3390/jcm9010022)
Supplement: Supplementary file 1 [file jcm-09-00022-s001.pdf]

Table 1. S1. Clinical factors associated with statin exposure.

Factor	Statin	Effect	References
		**Clinical**	
Advanced age	ATV, LVT, PIT, PVT, SVT,	-↑AUC: **ATV** (30%), **PIT** (30%), **PVT** (~37%)<br>-↑mean plasma level of HMG-CoA reductase inhibitory activity: **LVT** (45%), **SVT** (45%)	[1-5]
	FVT, RVT	No significant difference	[6,7]
Gender	ATV	Women: 20% ↑**ATV** C_{max}, 10% ↓AUC	[1]
	FVT, PIT	Women: ↑ **FVT** or **PIT** AUC	[6,8]
	PVT	Women: ↑ **PVT** AUC (in subjects homozygous wild type at *SLCO1B1* rs4149056)	[9]
	RVT	No significant difference	[7,10]
	SVT	- **SVT acid**: ↑ exposure in women<br>- SVT: no significant difference	[11]
Dose	ATV, FVT, LVT, PIT, PVT,RVT, SVT,	↑ statin plasma exposure	[1-7]
Evening compared to morning dosing	ATV, PVT	- **ATV** AUC ↓ by 29%<br>- **PVT** bioavailability ↓ by 60%	[1,4,12]
	PIT, RVT	No significant difference in AUC	[3,7,13]
		Ethnicity (compared to Caucasian)	
African American	PVT	1.4 fold ↑ **PVT** AUC	[14]
Asian	ATV	No significant difference in ATV AUC	[15]
Asian-Indian	RVT	26% non-significant ↑ in RVT AUC	[16]
East Asian	RVT	64-84% ↑ **RVT** AUC	[16]
Japanese	RVT, SVT, PIT	- 55% ↑ exposure to **RVT**<br>- 34-68% ↑ exposure to **SVT acid**<br>- no significant difference for PIT	[3,17,18]
Mexican	PVT	No significant difference in AUC	[19]
Alcohol	FVT	- 30% ↑ **FVT** AUC in chronic alcohol consumption<br>- no significant difference in FVT AUC in acute alcohol consumption:	[20]
Co-administration with food	LVT	~50% ↑ **LVT** AUC	[2]
	PVT	↓ **PVT** C_{max} (by 49%) and AUC (by 31%)	[21]
	ATV, FVT, PIT	- Significant ↓ in C_{max} (**ATV** by 47.9%, **FVT** by 40-60%, PIT by 43%)<br>- Non-significant ↓ in AUC (ATV 9-13%, FVT by 11%)	[3,6,22,23]

	RVT, SVT	No significant difference in AUC	[5,7]
Diabetes mellitus	ATV	↑ plasma concentrations of ATV and metabolites when sampled >5-24 hours post dose	[24]
Hepatic impairment	ATV, FVT, PIT, PVT	↑ AUC: **ATV** (4-11 fold), **FVT** (2.5 fold), **PIT** (1.6-3.8 fold) **PVT** (34%)	[1,3,6,25]
	RVT	No significant difference	[26]
Renal impairment	LVT, PIT, PVT, RVT	↑ AUC: **LVT** (2 fold), **PIT** (2 fold), **PVT** (69%), **RVT** (3 fold)	[2-4,7]
	ATV, FVT	No significant difference	[1,27]
	SVT	Potentially no difference	[28]
Following bariatric surgery	ATV	- 2 fold ↑ **ATV** AUC 3-8 weeks after surgery<br>-↓ **ATV** AUC ~2 years after surgery	[29]
		**Selected co-medications**	
		Strong CYP3A4 inhibitors	
Itraconazole	ATV, LVT, PVT, RVT, SVT	↑ AUC: **ATV** (3.3 fold), **LVT** (>15-20 fold), **LVT acid** (15-20 fold), **PVT** (11-72%), **RVT** (28-39%), **SVT** (10 fold), **SVT acid** (19 fold)	[1,4,30-34]
	PIT	**PIT** AUC ↓ by 23%	[35]
	FVT	No significant difference	[34]
Ketoconazole	RVT	No significant difference	[36]
Clarithromycin	ATV, PVT, SVT	↑ AUC: **ATV** (4.4. fold), **PVT** (2.1 fold), **SVT** (10 fold), **SVT acid** (12 fold)	[1,4,37]
		Moderate CYP3A4 inhibitors	
Erythromycin	ATV, PIT, SVT	↑ AUC: **ATV** (33%), **PIT** (2.8 fold), **SVT** (6.2 fold), **SVT acid** (3.9 fold)	[1,3,38,39]
	RVT	**RVT** AUC ↓ by 20%	[40]
	FVT	No significant difference	[20]
Grapefruit juice (low dose)	ATV, LVT, PIT, SVT	↑ AUC: **ATV** (40-83%), **LVT** (1.94 fold), **PIT** (14%), **SVT** (1.9-3.6 fold), **SVT acid** (1.3-3.3 fold)	[5,41-45]
	PVT	No significant difference	[42]
Diltiazem	ATV, LVT, SVT	↑ AUC: **ATV** (51%), **LVT** (3.57 fold), **SVT** (3.10-4.6 fold), **SVT acid** (2.69 fold)	[1,2,5]
	PIT	10% ↑ PIT AUC, but statistical significance unknown	[3]
	PVT	No significant difference	[4]
		Weak CYP3A4 inhibitors	
Amlodipine	ATV, SVT	↑ AUC: **ATV** (18%), **SVT** (1.77 fold), **SVT acid** (1.58 fold)	[5,46]
	FVT	No significant different	[47]
		Other drugs	
Ciclosporin (inhibits CYP3A4, OATP1B1/1B3/2B1, ABCG2, P-gp)	ATV, FVT, LVT, PIT, PVT, RVT, SVT	↑ AUC: **ATV** (8.7 fold), **FVT** (90%), **LVT** (5-8 fold), **PIT** (4.6 fold), **PVT** (3.8 fold), **RVT** (7 fold), **SVT** (8 fold)	[1-7,48]

Fluconazole (inhibits CYP2C9, 2C19 & 3A)	FVT	84% ↑ **FVT** AUC	[49]
	PVT, RVT	No significant difference	[49,50]
Gemfibrozil (inhibits CYP2C8, OATP1B1 & OAT3)	ATV, LVT acid, PIT, PVT, RVT, SVT	↑ AUC: **ATV** (35%), **LVT acid** (2.8 fold), LVT (no significant difference), **PIT** (45%), **PVT** (2 fold), **RVT** (1.9 fold), **SVT** (1.35 fold), **SVT acid** (2.85 fold)	[51-56]
	FVT	No significant difference	[56,57]
Letermovir (inhibits OATP1B1/1B3 & CYP3A)	ATV	- 3 fold ↑ **ATV** AUC	[58]
Rifampicin (induces CYP3A; inhibits OATP1B1)	ATV	- 7 fold ↑ **ATV** AUC if single rifampicin dose co-administered with a single ATV dose<br>- 30% ↑ **ATV** AUC if ATV/rifampicin co-administered after a 5 day course of rifampicin<br>- **ATV** AUC ↓ by 80% if ATV given separately after a 5 day course of rifampicin	[1,59,60]
	PIT	6.7 fold ↑ **PIT** AUC if single rifampicin dose co-administered with a single PIT dose	[61]
	PVT	- 2.3 fold ↑ **PVT** AUC if single rifampicin dose co-administered with a single PVT dose<br>- **PVT** AUC ↓ by 31% if PVT given separately after a 5 day course of rifampicin	[62,63]
	FVT, SVT	↓ AUC if statin given separately following rifampicin pre-treatment: **FVT** (by 53%), **SVT** (by 87%), **SVT acid** (by 93%). (Probable ↓ in LVT AUC)	[6,64,65]
	RVT	No significant difference when RVT given separately after a 6 day course of rifampicin	[66]
Tocilizumab (inhibits IL-6R)	SVT	**SVT** AUC ↓ by 57%	[67]

Unless otherwise stated, reported changes were statistically significant (statin highlighted in bold font).
AUC = area under the concentration-time curve.

Table S2. Genetic variants associated with statin exposure

Gene	Variant	Comparison	Statin	Effect	References
SLCO1B1	rs4149056 (521T>C, p.V174A)	CC vs TT	ATV	- ↑ AUC: **ATV** (2.44 fold, 144%), **2-OH ATV** (2.0 fold)<br>- Non-significant ↑ in AUC: ATV L (2.0 fold), 2-OH ATV L (1.6 fold)	[68]
			FVT	1.19 (19%) fold non-significant ↑ in FVT AUC	[9]
			LVT	- 3.86 fold (286%) ↑ **LVT acid** AUC<br>- LVT AUC ↓ non-significantly by 16%	[69]
			PIT	- 3.08 fold (208%) ↑ **PIT** AUC<br>- No significant difference in PIT L AUC	[70]
			PVT	- 1.91 (91%) fold ↑ **PVT** AUC	[9]
			RVT	- 1.65 (65%) fold ↑ **RVT** AUC	[68]
			SVT	- 3.21 (221%) fold ↑ **SVT acid** AUC<br>- 1.43 fold non-significant ↑ in SVT AUC	[71]
	rs2306283 (388A>G, p.N130D)	Haplotype **14* (rs2306283 but not rs4149056) vs **1a*	ATV	↓ **ATV** AUC	[72]
		GG (**1b/*1b*) vs AA (**1a/*1a*)	LVT	- **LVT acid** AUC ↓ by 32%<br>- LVT AUC ↓ non-significantly by 28%	[69]
SLCO2B1	rs12422149 (935G>A, p.R312Q)	AA vs GG	SVT	79% ↑ apparent clearance of **SVT acid**	[17]
SLC10A1	rs2296651 (p.S267F, **2*)	**2* carriers vs **1/*1*	RVT	- 57% ↑ **RVT** AUC<br>- No significant differences in RVT or N-desmethyl RVT plasma concentrations	[73,74]
SLC22A8	rs2276299 (723T>A)	A carriers vs TT	PVT	No significant difference in renal clearance	[75]
	rs749911923 (1166C>T, p.A389V)	CT vs CC	PVT	No significant difference in renal clearance	[75]
ABCC2	rs113646094 (1446C>G)	GC vs CC	PVT	- ↓ PVT AUC by 68%	[76]
ABCB1	rs1128503-rs2032582-rs1045642 (1236C-T-2677G-T-3435C-T)	TTT/TTT vs CGC/CGC	ATV	- 55% ↑ **ATV** AUC<br>- No significant differences for ATV L	[77]
			SVT	- 60% ↑ **SVT acid** AUC<br>- No significant differences for SVT	[77]
			FVT	No significant differences	[78]
			LVT	No significant differences	[78]
			PVT	No significant differences	[78]
			RVT	No significant differences	[78]
	rs2032582-rs1045642 (2677G-T-3435C-T)	TT/TT vs GG/CC and GT/CT	ATV	- ↑ elimination $t_{1/2}$: **ATV**, **ATV L**, **2-OH ATV**, **2-OH ATV L**<br>- No significant differences in AUC	[79]

ABCB11	rs2287622 (1331T>C, p.V444A)	CC vs CT vs TT	PVT	No significant differences	[14]
	rs11568364 (2029A>G, p.M677V)	GG vs GA vs GG	PVT	No significant differences	[14]
ABCC2	rs717620 (-24C>T)	TT vs CT vs CC	PIT	- ↓ **PIT** AUC<br>- **PIT** AUC ↓ by 62% in TT compared to CC subjects	[80]
	rs113646094 (1446C>G, p.T482=)	CG vs CC	PVT	**PVT** AUC ↓ by 67%	[76]
ABCG2	rs2231142 (421C>A, p.Q141K)	AA vs CC	ATV	- ↑ AUC: **ATV** (1.7 fold), **ATV L** (1.9 fold)<br>- Non-significant ↑ in AUC: 2-OH ATV (1.4 fold), 2-OH ATV L (1.5 fold), 4-OH ATV L (1.4 fold)	[81]
			FVT	1.7 fold ↑ **FVT** AUC	[82]
			PIT	No significant differences	[80,83]
			PVT	No significant differences	[82]
			RVT	- 2.4 fold ↑ **RVT** AUC<br>-2 fold ↑ in **N-desmethyl RVT** plasma concentration	[73,81]
			SVT	- 2.1 fold ↑ **SVT** AUC<br>- 1.2 non-significant ↑ in SVT acid AUC	[82]
PPARA	rs4253728 (209-1003G>A)	GA vs GG	ATV	↓ **2-OH ATV** to ATV AUC ratio	[84]
		GA/AA vs GG	SVT	24% ↓ apparent clearance of **SVT acid**	[17]
CYP2C9	rs1799853 (p.R144C, **2*)	**2/*2* vs **1/*1*	FVT	No significant differences	[85]
	rs1057910 (p.I359L, **3*)	**3/*3* vs **1/*1*	FVT	3.1-5.0 fold ↑ in **FVT** AUC	[85]
		**3* carriers vs *1/*1*	PIT	- 2.9 fold ↑ in **PIT** AUC<br>- 1.7 fold ↑ in **PIT L** AUC	[83]
			RVT	No significant differences in RVT or N-desmethyl RVT plasma concentrations	[10,73]
			SVT	No significant differences	[86]
CYP2C19	- rs4244285 (p.P227=, **2*)<br>- rs4986893 (p.W212Stop gained, **3)*	**2/*2*, **2/*3* or **3/*3* (PMs) vs **1/*2*, **1/*3* or **1/*1* (EMs)	RVT	- No significant differences in RVT, N-desmethyl RVT or RVT L pharmacokinetics	[73,87]
CYP2D6	whole gene deletion (**5*)	**5/*5* vs *wt/wt* (**1* or **2*)	LVT	- 5.1 fold ↑ **LVT** AUC<br>- no significant difference in LVT acid AUC	[88]
		**5/wt* vs *wt/wt*	SVT	- **SVT** AUC ↓ by 23%<br>- no significant difference in SVT acid AUC	[89]
	rs1065852 (p.S34P, **10*)	**10/*10* vs *wt/wt* (**1* or **2*)	LVT	- 2.2 fold ↑ **LVT** AUC<br>- no significant difference in LVT acid AUC	[88]
	rs5030865 (p.G169Stop gained, **14*)	**14/*14* vs *wt/wt*	SVT	- 1.5 fold ↑ **SVT** AUC<br>- no significant difference in SVT acid AUC	[89]

	rs28371725 (**41*)	**41/*41* vs *wt/wt*	SVT	↑ C_{max} of both **SVT** and **SVT acid**	[89]
CYP3A4	rs35599367 (**22*)	**22* carriers vs **1/*1*	ATV	↓ **2-OH ATV** to ATV AUC ratio	[84]
			SVT	White patients:<br>- 14% ↑ **SVT acid** concentration<br>- 20% non-significantly ↑ SVT concentration<br>African American patients:<br>- 170% ↑ **SVT** concentration<br>- no significant difference in SVT acid concentration	[90]
CYP3A5	rs776746 (**3*)	**3/*3* vs **1* carriers	ATV	- 36% ↑ **ATV L** AUC<br>- No significant difference in ATV AUC	[91]
		**3/*3* vs **1/*3*	PIT	No significant differences for PIT or PIT L	[83]
		**3/*3* vs **1/*3*	RVT	No significant differences	[10]
		**3/*3* vs **1/*1*	SVT	- 3.3 fold ↑ **SVT** AUC<br>- No significant difference in SVT acid plasma levels	[90,92]
UGT1A1	**28*	**28* vs **1/*1*	ATV	- 41% ↓ **ATV L** AUC	[93]
UGT1A3	**2*	**2/*2* vs **1/*1*	ATV	- 72% ↑ **ATV L** AUC<br>- 160% ↑ **2OH ATV L** AUC	[94,95]
		**1/*2* vs **1/*1*	ATV	↑ **ATV L** to ATV AUC ratio	[94]

Unless otherwise stated, reported changes were statistically significant (statin highlighted in bold font).
AUC = area under the concentration-time curve; wt = wild type.

Supplementary References

1. Pfizer Inc. Lipitor- atorvastatin calcium trihydrate tablet, film coated. Highlights of prescribing information. http://labeling.pfizer.com/ShowLabeling.aspx?id=587 (13 May),
2. Merck & Co, I. Mevacor (lovastatin) tablets description. https://www.merck.com/product/usa/pi_circulars/m/mevacor/mevacor_pi.pdf (7 July),
3. Kowa Pharmaceuticals. Livalo (pitavastatin) tablet - highlights of prescribing information. http://www.accessdata.fda.gov/drugsatfda_docs/label/2012/022363s008s009lbl.pdf (7 July),
4. Bristol-Myers Squibb Company. Pravachol (pravastatin) tablets - highlights of prescribing information. http://packageinserts.bms.com/pi/pi_pravachol.pdf (7 July),
5. Merck & Co, I. Zocor (simvastatin) tablets - highlights of prescribing information. https://www.merck.com/product/usa/pi_circulars/z/zocor/zocor_pi.pdf (7 July),
6. Novartis. Lescol (fluvastatin dosium) - highlights of prescribing information. https://www.pharma.us.novartis.com/sites/www.pharma.us.novartis.com/files/Lescol.pdf (7 July),
7. AstraZeneca. Crestor (rosuvastatin calcium tablets) - highlights of prescribing information. http://www.accessdata.fda.gov/drugsatfda_docs/label/2010/021366s016lbl.pdf (3 October),
8. RxList. Livalo (pitavastatin). http://www.rxlist.com/livalo-drug/clinical-pharmacology.htm (7 July),
9. Niemi, M.; Pasanen, M.K.; Neuvonen, P.J. Slco1b1 polymorphism and sex affect the pharmacokinetics of pravastatin but not fluvastatin. *Clinical pharmacology and therapeutics* **2006**, *80*, 356-366.
10. Zhou, Q.; Ruan, Z.R.; Yuan, H.; Xu, D.H.; Zeng, S. Abcb1 gene polymorphisms, abcb1 haplotypes and abcg2 c.421c > a are determinants of inter-subject variability in rosuvastatin pharmacokinetics. *Die Pharmazie* **2013**, *68*, 129-134.
11. Yang, W.; Xu, H.; Song, Y.; Wang, X.; Ren, X.; Zhao, D.; Cai, Y.; Zhang, S.; Huang, J.; Zhang, L.R.*, et al.* Pharmacokinetics of simvastatin lactone and its active metabolite simvastatin hydroxy acid in healthy chinese male and female volunteers. *International journal of clinical pharmacology and therapeutics* **2014**, *52*, 151-158.
12. Cilla, D.D., Jr.; Gibson, D.M.; Whitfield, L.R.; Sedman, A.J. Pharmacodynamic effects and pharmacokinetics of atorvastatin after administration to normocholesterolemic subjects in the morning and evening. *Journal of clinical pharmacology* **1996**, *36*, 604-609.
13. Martin, P.D.; Mitchell, P.D.; Schneck, D.W. Pharmacodynamic effects and pharmacokinetics of a new hmg-coa reductase inhibitor, rosuvastatin, after morning or evening administration in healthy volunteers. *British journal of clinical pharmacology* **2002**, *54*, 472-477.
14. Ho, R.H.; Choi, L.; Lee, W.; Mayo, G.; Schwarz, U.I.; Tirona, R.G.; Bailey, D.G.; Stein, C.M.; Kim, R.B. Effect of drug transporter genotypes on pravastatin disposition in european- and african-american participants. *Pharmacogenetics and genomics* **2007**, *17*, 647-656.
15. Gandelman, K.; Fung, G.L.; Messig, M.; Laskey, R. Systemic exposure to atorvastatin between asian and caucasian subjects: A combined analysis of 22 studies. *American journal of therapeutics* **2012**, *19*, 164-173.
16. Birmingham, B.K.; Bujac, S.R.; Azumaya, C.T.; Zalikowski, J.; Chen, Y.; Kim, K.; Ambrose, H.J. Rosuvastatin pharmacokinetics and pharmacogenetics in caucasian and asian subjects residing in the united states. *European journal of clinical pharmacology* **2015**, *71*, 329-340.
17. Tsamandouras, N.; Dickinson, G.; Guo, Y.; Hall, S.; Rostami-Hodjegan, A.; Galetin, A.; Aarons, L. Identification of the effect of multiple polymorphisms on the pharmacokinetics of simvastatin and simvastatin acid using a population-modeling approach. *Clinical pharmacology and therapeutics* **2014**, *96*, 90-100.
18. Birmingham, B.K.; Bujac, S.R.; Elsby, R.; Azumaya, C.T.; Wei, C.; Chen, Y.; Mosqueda-Garcia, R.; Ambrose, H.J. Impact of abcg2 and slco1b1 polymorphisms on pharmacokinetics of rosuvastatin, atorvastatin and simvastatin acid in caucasian and asian subjects: A class effect? *European journal of clinical pharmacology* **2015**, *71*, 341-355.

19. Escobar, Y.; Venturelli, C.R.; Hoyo-Vadillo, C. Pharmacokinetic properties of pravastatin in mexicans: An open-label study in healthy adult volunteers. *Current therapeutic research, clinical and experimental* **2005**, *66*, 238-246.
20. Scripture, C.D.; Pieper, J.A. Clinical pharmacokinetics of fluvastatin. *Clinical pharmacokinetics* **2001**, *40*, 263-281.
21. Pan, H.Y.; DeVault, A.R.; Brescia, D.; Willard, D.A.; McGovern, M.E.; Whigan, D.B.; Ivashkiv, E. Effect of food on pravastatin pharmacokinetics and pharmacodynamics. *International journal of clinical pharmacology, therapy, and toxicology* **1993**, *31*, 291-294.
22. Radulovic, L.L.; Cilla, D.D.; Posvar, E.L.; Sedman, A.J.; Whitfield, L.R. Effect of food on the bioavailability of atorvastatin, an hmg-coa reductase inhibitor. *Journal of clinical pharmacology* **1995**, *35*, 990-994.
23. Smith, H.T.; Jokubaitis, L.A.; Troendle, A.J.; Hwang, D.S.; Robinson, W.T. Pharmacokinetics of fluvastatin and specific drug interactions. *American journal of hypertension* **1993**, *6*, 375s-382s.
24. Dostalek, M.; Sam, W.J.; Paryani, K.R.; Macwan, J.S.; Gohh, R.Y.; Akhlaghi, F. Diabetes mellitus reduces the clearance of atorvastatin lactone: Results of a population pharmacokinetic analysis in renal transplant recipients and in vitro studies using human liver microsomes. *Clinical pharmacokinetics* **2012**, *51*, 591-606.
25. Hatanaka, T. Clinical pharmacokinetics of pravastatin: Mechanisms of pharmacokinetic events. *Clinical pharmacokinetics* **2000**, *39*, 397-412.
26. Simonson, S.G.; Martin, P.D.; Mitchell, P.; Schneck, D.W.; Lasseter, K.C.; Warwick, M.J. Pharmacokinetics and pharmacodynamics of rosuvastatin in subjects with hepatic impairment. *European journal of clinical pharmacology* **2003**, *58*, 669-675.
27. Appel-Dingemanse, S.; Smith, T.; Merz, M. Pharmacokinetics of fluvastatin in subjects with renal impairment and nephrotic syndrome. *Journal of clinical pharmacology* **2002**, *42*, 312-318.
28. Launay-Vacher, V.; Izzedine, H.; Deray, G. Statins' dosage in patients with renal failure and cyclosporine drug-drug interactions in transplant recipient patients. *International journal of cardiology* **2005**, *101*, 9-17.
29. Jakobsen, G.S.; Skottheim, I.B.; Sandbu, R.; Christensen, H.; Roislien, J.; Asberg, A.; Hjelmesaeth, J. Long-term effects of gastric bypass and duodenal switch on systemic exposure of atorvastatin. *Surgical endoscopy* **2013**, *27*, 2094-2101.
30. Cooper, K.J.; Martin, P.D.; Dane, A.L.; Warwick, M.J.; Schneck, D.W.; Cantarini, M.V. Effect of itraconazole on the pharmacokinetics of rosuvastatin. *Clinical pharmacology and therapeutics* **2003**, *73*, 322-329.
31. Neuvonen, P.J.; Jalava, K.M. Itraconazole drastically increases plasma concentrations of lovastatin and lovastatin acid. *Clinical pharmacology and therapeutics* **1996**, *60*, 54-61.
32. Neuvonen, P.J.; Kantola, T.; Kivisto, K.T. Simvastatin but not pravastatin is very susceptible to interaction with the cyp3a4 inhibitor itraconazole. *Clinical pharmacology and therapeutics* **1998**, *63*, 332-341.
33. Mazzu, A.L.; Lasseter, K.C.; Shamblen, E.C.; Agarwal, V.; Lettieri, J.; Sundaresen, P. Itraconazole alters the pharmacokinetics of atorvastatin to a greater extent than either cerivastatin or pravastatin. *Clinical pharmacology and therapeutics* **2000**, *68*, 391-400.
34. Kivisto, K.T.; Kantola, T.; Neuvonen, P.J. Different effects of itraconazole on the pharmacokinetics of fluvastatin and lovastatin. *British journal of clinical pharmacology* **1998**, *46*, 49-53.
35. Nakagawa, S.; Gosho, M.; Inazu, Y.; Hounslow, N. Pitavastatin concentrations are not increased by cyp3a4 inhibitor itraconazole in healthy subjects. *Clinical pharmacology in drug development* **2013**, *2*, 195-200.
36. Cooper, K.J.; Martin, P.D.; Dane, A.L.; Warwick, M.J.; Raza, A.; Schneck, D.W. Lack of effect of ketoconazole on the pharmacokinetics of rosuvastatin in healthy subjects. *British journal of clinical pharmacology* **2003**, *55*, 94-99.
37. Jacobson, T.A. Comparative pharmacokinetic interaction profiles of pravastatin, simvastatin, and atorvastatin when coadministered with cytochrome p450 inhibitors. *The American journal of cardiology* **2004**, *94*, 1140-1146.

38. Kantola, T.; Kivisto, K.T.; Neuvonen, P.J. Erythromycin and verapamil considerably increase serum simvastatin and simvastatin acid concentrations. *Clinical pharmacology and therapeutics* **1998**, *64*, 177-182.
39. Siedlik, P.H.; Olson, S.C.; Yang, B.B.; Stern, R.H. Erythromycin coadministration increases plasma atorvastatin concentrations. *Journal of clinical pharmacology* **1999**, *39*, 501-504.
40. Cooper, K.J.; Martin, P.D.; Dane, A.L.; Warwick, M.J.; Raza, A.; Schneck, D.W. The effect of erythromycin on the pharmacokinetics of rosuvastatin. *European journal of clinical pharmacology* **2003**, *59*, 51-56.
41. Ando, H.; Tsuruoka, S.; Yanagihara, H.; Sugimoto, K.; Miyata, M.; Yamazoe, Y.; Takamura, T.; Kaneko, S.; Fujimura, A. Effects of grapefruit juice on the pharmacokinetics of pitavastatin and atorvastatin. *British journal of clinical pharmacology* **2005**, *60*, 494-497.
42. Fukazawa, I.; Uchida, N.; Uchida, E.; Yasuhara, H. Effects of grapefruit juice on pharmacokinetics of atorvastatin and pravastatin in japanese. *British journal of clinical pharmacology* **2004**, *57*, 448-455.
43. Lilja, J.J.; Neuvonen, M.; Neuvonen, P.J. Effects of regular consumption of grapefruit juice on the pharmacokinetics of simvastatin. *British journal of clinical pharmacology* **2004**, *58*, 56-60.
44. Rogers, J.D.; Zhao, J.; Liu, L.; Amin, R.D.; Gagliano, K.D.; Porras, A.G.; Blum, R.A.; Wilson, M.F.; Stepanavage, M.; Vega, J.M. Grapefruit juice has minimal effects on plasma concentrations of lovastatin-derived 3-hydroxy-3-methylglutaryl coenzyme a reductase inhibitors. *Clinical pharmacology and therapeutics* **1999**, *66*, 358-366.
45. Hu, M.; Mak, V.W.; Yin, O.Q.; Chu, T.T.; Tomlinson, B. Effects of grapefruit juice and slco1b1 388a>g polymorphism on the pharmacokinetics of pitavastatin. *Drug metabolism and pharmacokinetics* **2013**, *28*, 104-108.
46. Drugs.com. Amlodipine and atorvastatin tablets. https://www.drugs.com/pro/amlodipine-and-atorvastatin-tablets.html (12 July),
47. Prasad, P.P.; Stypinski, D.; Vyas, K.H.; Gonasun, L. Lack of interaction between modified-release fluvastatin and amlodipine in healthy subjects. *Clinical drug investigation* **2004**, *24*, 323-331.
48. Ichimaru, N.; Takahara, S.; Kokado, Y.; Wang, J.D.; Hatori, M.; Kameoka, H.; Inoue, T.; Okuyama, A. Changes in lipid metabolism and effect of simvastatin in renal transplant recipients induced by cyclosporine or tacrolimus. *Atherosclerosis* **2001**, *158*, 417-423.
49. Kantola, T.; Backman, J.T.; Niemi, M.; Kivisto, K.T.; Neuvonen, P.J. Effect of fluconazole on plasma fluvastatin and pravastatin concentrations. *European journal of clinical pharmacology* **2000**, *56*, 225-229.
50. Cooper, K.J.; Martin, P.D.; Dane, A.L.; Warwick, M.J.; Schneck, D.W.; Cantarini, M.V. The effect of fluconazole on the pharmacokinetics of rosuvastatin. *European journal of clinical pharmacology* **2002**, *58*, 527-531.
51. Kyrklund, C.; Backman, J.T.; Neuvonen, M.; Neuvonen, P.J. Gemfibrozil increases plasma pravastatin concentrations and reduces pravastatin renal clearance. *Clinical pharmacology and therapeutics* **2003**, *73*, 538-544.
52. Whitfield, L.R.; Porcari, A.R.; Alvey, C.; Abel, R.; Bullen, W.; Hartman, D. Effect of gemfibrozil and fenofibrate on the pharmacokinetics of atorvastatin. *Journal of clinical pharmacology* **2011**, *51*, 378-388.
53. Matthew, P.; Cuddy, R.; Tracewell, W.G.; Salazar, D. An open-label study on the pharmacokinetics (pk) of pitavastatin (nk-104) when administered concomitantly with fenofibrate or gemfibrozil in healthy volunteers (abstract). *Clinical pharmacology and therapeutics* **2004**, *75*, 33.
54. Schneck, D.W.; Birmingham, B.K.; Zalikowski, J.A.; Mitchell, P.D.; Wang, Y.; Martin, P.D.; Lasseter, K.C.; Brown, C.D.; Windass, A.S.; Raza, A. The effect of gemfibrozil on the pharmacokinetics of rosuvastatin. *Clinical pharmacology and therapeutics* **2004**, *75*, 455-463.
55. Backman, J.T.; Kyrklund, C.; Kivisto, K.T.; Wang, J.S.; Neuvonen, P.J. Plasma concentrations of active simvastatin acid are increased by gemfibrozil. *Clinical pharmacology and therapeutics* **2000**, *68*, 122-129.

56. Kyrklund, C.; Backman, J.T.; Kivisto, K.T.; Neuvonen, M.; Laitila, J.; Neuvonen, P.J. Plasma concentrations of active lovastatin acid are markedly increased by gemfibrozil but not by bezafibrate. *Clinical pharmacology and therapeutics* **2001**, *69*, 340-345.
57. Spence, J.D.; Munoz, C.E.; Hendricks, L.; Latchinian, L.; Khouri, H.E. Pharmacokinetics of the combination of fluvastatin and gemfibrozil. *The American journal of cardiology* **1995**, *76*, 80a-83a.
58. Merck Sharp & Dohme Corp. Prevymis (letermovir) highlights of prescribing information. https://www.accessdata.fda.gov/drugsatfda_docs/label/2017/209939Orig1s000,209940Orig1s000lbl.pdf (07 November),
59. Backman, J.T.; Luurila, H.; Neuvonen, M.; Neuvonen, P.J. Rifampin markedly decreases and gemfibrozil increases the plasma concentrations of atorvastatin and its metabolites. *Clinical pharmacology and therapeutics* **2005**, *78*, 154-167.
60. Rhoda Lee, C.; Sharp, J.; Baxter, K. Drug interactions: Cytochrome p450 isoenzymes not the only interaction mechanism. http://www.pharmaceutical-journal.com/learning/learning-article/drug-interactions-cytochrome-p450-isoenzymes-not-the-only-interaction-mechanism/11098984.article (12 July),
61. Chen, Y.; Zhang, W.; Huang, W.H.; Tan, Z.R.; Wang, Y.C.; Huang, X.; Zhou, H.H. Effect of a single-dose rifampin on the pharmacokinetics of pitavastatin in healthy volunteers. *European journal of clinical pharmacology* **2013**, *69*, 1933-1938.
62. Deng, S.; Chen, X.P.; Cao, D.; Yin, T.; Dai, Z.Y.; Luo, J.; Tang, L.; Li, Y.J. Effects of a concomitant single oral dose of rifampicin on the pharmacokinetics of pravastatin in a two-phase, randomized, single-blind, placebo-controlled, crossover study in healthy chinese male subjects. *Clinical therapeutics* **2009**, *31*, 1256-1263.
63. Kyrklund, C.; Backman, J.T.; Neuvonen, M.; Neuvonen, P.J. Effect of rifampicin on pravastatin pharmacokinetics in healthy subjects. *British journal of clinical pharmacology* **2004**, *57*, 181-187.
64. Kyrklund, C.; Backman, J.T.; Kivisto, K.T.; Neuvonen, M.; Laitila, J.; Neuvonen, P.J. Rifampin greatly reduces plasma simvastatin and simvastatin acid concentrations. *Clinical pharmacology and therapeutics* **2000**, *68*, 592-597.
65. Neuvonen, P.J.; Backman, J.T.; Niemi, M. Pharmacokinetic comparison of the potential over-the-counter statins simvastatin, lovastatin, fluvastatin and pravastatin. *Clinical pharmacokinetics* **2008**, *47*, 463-474.
66. Zhang, W.; Deng, S.; Chen, X.P.; Zhou, G.; Xie, H.T.; He, F.Y.; Cao, D.; Li, Y.J.; Zhou, H.H. Pharmacokinetics of rosuvastatin when coadministered with rifampicin in healthy males: A randomized, single-blind, placebo-controlled, crossover study. *Clinical therapeutics* **2008**, *30*, 1283-1289.
67. Schmitt, C.; Kuhn, B.; Zhang, X.; Kivitz, A.J.; Grange, S. Disease-drug-drug interaction involving tocilizumab and simvastatin in patients with rheumatoid arthritis. *Clinical pharmacology and therapeutics* **2011**, *89*, 735-740.
68. Pasanen, M.K.; Fredrikson, H.; Neuvonen, P.J.; Niemi, M. Different effects of slco1b1 polymorphism on the pharmacokinetics of atorvastatin and rosuvastatin. *Clinical pharmacology and therapeutics* **2007**, *82*, 726-733.
69. Tornio, A.; Vakkilainen, J.; Neuvonen, M.; Backman, J.T.; Neuvonen, P.J.; Niemi, M. Slco1b1 polymorphism markedly affects the pharmacokinetics of lovastatin acid. *Pharmacogenetics and genomics* **2015**.
70. Ieiri, I.; Suwannakul, S.; Maeda, K.; Uchimaru, H.; Hashimoto, K.; Kimura, M.; Fujino, H.; Hirano, M.; Kusuhara, H.; Irie, S., *et al.* Slco1b1 (oatp1b1, an uptake transporter) and abcg2 (bcrp, an efflux transporter) variant alleles and pharmacokinetics of pitavastatin in healthy volunteers. *Clinical pharmacology and therapeutics* **2007**, *82*, 541-547.
71. Pasanen, M.K.; Neuvonen, M.; Neuvonen, P.J.; Niemi, M. Slco1b1 polymorphism markedly affects the pharmacokinetics of simvastatin acid. *Pharmacogenetics and genomics* **2006**, *16*, 873-879.
72. Nies, A.T.; Niemi, M.; Burk, O.; Winter, S.; Zanger, U.M.; Stieger, B.; Schwab, M.; Schaeffeler, E. Genetics is a major determinant of expression of the human hepatic uptake transporter oatp1b1, but not of oatp1b3 and oatp2b1. *Genome medicine* **2013**, *5*, 1.

73. Lee, H.K.; Hu, M.; Lui, S.; Ho, C.S.; Wong, C.K.; Tomlinson, B. Effects of polymorphisms in abcg2, slco1b1, slc10a1 and cyp2c9/19 on plasma concentrations of rosuvastatin and lipid response in chinese patients. *Pharmacogenomics* **2013**, *14*, 1283-1294.
74. Lou, X.Y.; Zhang, W.; Wang, G.; Hu, D.L.; Guo, D.; Tan, Z.R.; Zhou, H.H.; Chen, Y.; Bao, H.H. The effect of na+/taurocholate cotransporting polypeptide (ntcp) c.800c > t polymorphism on rosuvastatin pharmacokinetics in chinese healthy males. *Die Pharmazie* **2014**, *69*, 775-779.
75. Nishizato, Y.; Ieiri, I.; Suzuki, H.; Kimura, M.; Kawabata, K.; Hirota, T.; Takane, H.; Irie, S.; Kusuhara, H.; Urasaki, Y.*, et al.* Polymorphisms of oatp-c (slc21a6) and oat3 (slc22a8) genes: Consequences for pravastatin pharmacokinetics. *Clinical pharmacology and therapeutics* **2003**, *73*, 554-565.
76. Niemi, M.; Arnold, K.A.; Backman, J.T.; Pasanen, M.K.; Godtel-Armbrust, U.; Wojnowski, L.; Zanger, U.M.; Neuvonen, P.J.; Eichelbaum, M.; Kivisto, K.T.*, et al.* Association of genetic polymorphism in abcc2 with hepatic multidrug resistance-associated protein 2 expression and pravastatin pharmacokinetics. *Pharmacogenetics and genomics* **2006**, *16*, 801-808.
77. Keskitalo, J.E.; Kurkinen, K.J.; Neuvoneni, P.J.; Niemi, M. Abcb1 haplotypes differentially affect the pharmacokinetics of the acid and lactone forms of simvastatin and atorvastatin. *Clinical pharmacology and therapeutics* **2008**, *84*, 457-461.
78. Keskitalo, J.E.; Kurkinen, K.J.; Neuvonen, M.; Backman, J.T.; Neuvonen, P.J.; Niemi, M. No significant effect of abcb1 haplotypes on the pharmacokinetics of fluvastatin, pravastatin, lovastatin, and rosuvastatin. *British journal of clinical pharmacology* **2009**, *68*, 207-213.
79. Lee, Y.J.; Lee, M.G.; Lim, L.A.; Jang, S.B.; Chung, J.Y. Effects of slco1b1 and abcb1 genotypes on the pharmacokinetics of atorvastatin and 2-hydroxyatorvastatin in healthy korean subjects. *International journal of clinical pharmacology and therapeutics* **2010**, *48*, 36-45.
80. Oh, E.S.; Kim, C.O.; Cho, S.K.; Park, M.S.; Chung, J.Y. Impact of abcc2, abcg2 and slco1b1 polymorphisms on the pharmacokinetics of pitavastatin in humans. *Drug metabolism and pharmacokinetics* **2013**, *28*, 196-202.
81. Keskitalo, J.E.; Zolk, O.; Fromm, M.F.; Kurkinen, K.J.; Neuvonen, P.J.; Niemi, M. Abcg2 polymorphism markedly affects the pharmacokinetics of atorvastatin and rosuvastatin. *Clinical pharmacology and therapeutics* **2009**, *86*, 197-203.
82. Keskitalo, J.E.; Pasanen, M.K.; Neuvonen, P.J.; Niemi, M. Different effects of the abcg2 c.421c>a snp on the pharmacokinetics of fluvastatin, pravastatin and simvastatin. *Pharmacogenomics* **2009**, *10*, 1617-1624.
83. Zhou, Q.; Chen, Q.X.; Ruan, Z.R.; Yuan, H.; Xu, H.M.; Zeng, S. Cyp2c9*3(1075a > c), abcb1 and slco1b1 genetic polymorphisms and gender are determinants of inter-subject variability in pitavastatin pharmacokinetics. *Die Pharmazie* **2013**, *68*, 187-194.
84. Klein, K.; Thomas, M.; Winter, S.; Nussler, A.K.; Niemi, M.; Schwab, M.; Zanger, U.M. Ppara: A novel genetic determinant of cyp3a4 in vitro and in vivo. *Clinical pharmacology and therapeutics* **2012**, *91*, 1044-1052.
85. Kirchheiner, J.; Kudlicz, D.; Meisel, C.; Bauer, S.; Meineke, I.; Roots, I.; Brockmoller, J. Influence of cyp2c9 polymorphisms on the pharmacokinetics and cholesterol-lowering activity of (-)-3s,5r-fluvastatin and (+)-3r,5s-fluvastatin in healthy volunteers. *Clinical pharmacology and therapeutics* **2003**, *74*, 186-194.
86. Zhou, Q.; Ruan, Z.R.; Jiang, B.; Yuan, H.; Zeng, S. Simvastatin pharmacokinetics in healthy chinese subjects and its relations with cyp2c9, cyp3a5, abcb1, abcg2 and slco1b1 polymorphisms. *Die Pharmazie* **2013**, *68*, 124-128.
87. Finkelman, R.D.; Wang, T.D.; Wang, Y.; Azumaya, C.T.; Birmingham, B.K.; Wissmar, J.; Mosqueda-Garcia, R. Effect of cyp2c19 polymorphism on the pharmacokinetics of rosuvastatin in healthy taiwanese subjects. *Clinical pharmacology in drug development* **2015**, *4*, 33-40.
88. Yin, O.Q.; Mak, V.W.; Hu, M.; Fok, B.S.; Chow, M.S.; Tomlinson, B. Impact of cyp2d6 polymorphisms on the pharmacokinetics of lovastatin in chinese subjects. *European journal of clinical pharmacology* **2012**, *68*, 943-949.

89. Choi, H.Y.; Bae, K.S.; Cho, S.H.; Ghim, J.L.; Choe, S.; Jung, J.A.; Jin, S.J.; Kim, H.S.; Lim, H.S. Impact of cyp2d6, cyp3a5, cyp2c19, cyp2a6, slco1b1, abcb1, and abcg2 gene polymorphisms on the pharmacokinetics of simvastatin and simvastatin acid. *Pharmacogenetics and genomics* **2015**, *25*, 595-608.
90. Kitzmiller, J.P.; Luzum, J.A.; Baldassarre, D.; Krauss, R.M.; Medina, M.W. Cyp3a4*22 and cyp3a5*3 are associated with increased levels of plasma simvastatin concentrations in the cholesterol and pharmacogenetics study cohort. *Pharmacogenetics and genomics* **2014**, *24*, 486-491.
91. Shin, J.; Pauly, D.F.; Pacanowski, M.A.; Langaee, T.; Frye, R.F.; Johnson, J.A. Effect of cytochrome p450 3a5 genotype on atorvastatin pharmacokinetics and its interaction with clarithromycin. *Pharmacotherapy* **2011**, *31*, 942-950.
92. Kim, K.A.; Park, P.W.; Lee, O.J.; Kang, D.K.; Park, J.Y. Effect of polymorphic cyp3a5 genotype on the single-dose simvastatin pharmacokinetics in healthy subjects. *Journal of clinical pharmacology* **2007**, *47*, 87-93.
93. Stormo, C.; Bogsrud, M.P.; Hermann, M.; Asberg, A.; Piehler, A.P.; Retterstol, K.; Kringen, M.K. Ugt1a1*28 is associated with decreased systemic exposure of atorvastatin lactone. *Molecular diagnosis & therapy* **2013**, *17*, 233-237.
94. Riedmaier, S.; Klein, K.; Hofmann, U.; Keskitalo, J.E.; Neuvonen, P.J.; Schwab, M.; Niemi, M.; Zanger, U.M. Udp-glucuronosyltransferase (ugt) polymorphisms affect atorvastatin lactonization in vitro and in vivo. *Clinical pharmacology and therapeutics* **2010**, *87*, 65-73.
95. Cho, S.K.; Oh, E.S.; Park, K.; Park, M.S.; Chung, J.Y. The ugt1a3*2 polymorphism affects atorvastatin lactonization and lipid-lowering effect in healthy volunteers. *Pharmacogenetics and genomics* **2012**, *22*, 598-605.
